# Modelling respiratory syncytial virus age-specific risk of hospitalisation in term and preterm infants

**DOI:** 10.1186/s12879-024-09400-2

**Published:** 2024-05-21

**Authors:** Fiona Giannini, Alexandra B. Hogan, Mohinder Sarna, Kathryn Glass, Hannah C. Moore

**Affiliations:** 1 Wesfarmers Centre of Vaccines and Infectious Diseases, Telethon Kids Institute, University of Western Australia, 15 Hospital Ave, Nedlands, WA 6009 Australia; 2 School of Population Health, The University of New South Wales, Sydney, NSW 2052 Australia; 3 National Centre for Epidemiology and Population Health, The Australian National University, 62 Mills Rd, Acton ACT, 2601 Australia; 4 School of Population Health, Curtin University, Perth, WA 6002 Australia

**Keywords:** Respiratory Syncytial Virus, RSV, Mathematical model, Compartmental model, Infectious disease modelling, Transmission model

## Abstract

**Background:**

Respiratory syncytial virus (RSV) is the most common cause of acute lower respiratory infections in children worldwide. The highest incidence of severe disease is in the first 6 months of life, with infants born preterm at greatest risk for severe RSV infections. The licensure of new RSV therapeutics (a long-acting monoclonal antibody and a maternal vaccine) in Europe, USA, UK and most recently in Australia, has driven the need for strategic decision making on the implementation of RSV immunisation programs. Data driven approaches, considering the local RSV epidemiology, are critical to advise on the optimal use of these therapeutics for effective RSV control.

**Methods:**

We developed a dynamic compartmental model of RSV transmission fitted to individually-linked population-based laboratory, perinatal and hospitalisation data for 2000–2012 from metropolitan Western Australia (WA), stratified by age and prior exposure. We account for the differential risk of RSV-hospitalisation in full-term and preterm infants (defined as < 37 weeks gestation). We formulated a function relating age, RSV exposure history, and preterm status to the risk of RSV-hospitalisation given infection.

**Results:**

The age-to-risk function shows that risk of hospitalisation, given RSV infection, declines quickly in the first 12 months of life for all infants and is 2.6 times higher in preterm compared with term infants. The hospitalisation risk, given infection, declines to < 10% of the risk at birth by age 7 months for term infants and by 9 months for preterm infants.

**Conclusions:**

The dynamic model, using the age-to-risk function, characterises RSV epidemiology for metropolitan WA and can now be extended to predict the impact of prevention measures. The stratification of the model by preterm status will enable the comparative assessment of potential strategies in the extended model that target this RSV risk group relative to all-population approaches. Furthermore, the age-to-risk function developed in this work has wider relevance to the epidemiological characterisation of RSV.

**Supplementary Information:**

The online version contains supplementary material available at 10.1186/s12879-024-09400-2.

## Background

Respiratory syncytial virus (RSV) is the most common cause of acute lower respiratory infections (ALRI) and the leading cause of pneumonia in children worldwide [1]. Global 2019 estimates show RSV is responsible for > 3.6 million hospitalisations and > 101,000 deaths annually in children < 5 years [2]. RSV infections impact people of all ages, and due to imperfect immunity, repeat infection can occur throughout life. Almost all children will have experienced an RSV infection by the age of two years [2]. The highest incidence of severe disease is in young infants, with recent global estimates reporting that the hospitalisation rate for RSV-associated ALRI peaks in children aged 0–3 months, and 39% of RSV-associated ALRI hospitalisations occur in the first 6 months of life [2]. Of these infants, those born preterm are at greatest risk for severe RSV illness and complications [3]. The risk of RSV-hospitalisation is 2.9 times higher in infants born at 29–34 weeks gestation compared with term births [4]. With the proportion of preterm infants increasing worldwide (∼ 11% of births [5, 6]), and > 2 million infants annually born very preterm (< 32 weeks [7]), these children represent a significant risk group. To date, RSV mathematical models have not explicitly modelled the differential risk of severe RSV illness in preterm infants or any other sub-population [8].

In the last two years, the RSV prevention landscape has changed dramatically through the inclusion of > 30 RSV prevention candidates across Phase 1, 2, and 3 trials [9], with some products, including a long-acting monoclonal antibody, now licensed [10] and in use. With RSV immunisation on the horizon, data-driven approaches to country- and region-specific vaccine policy design and implementation are needed. Mathematical models for infectious diseases are key tools for understanding transmission, mitigating healthcare system impact, and planning the use of new therapeutics and interventions, and hence are valuable for the development of effective policy. For mathematical models to inform the implementation of immunisation strategies, highrisk groups including those born preterm, need to be considered to capture the full cost and impact of immunisation.

One aspect of RSV epidemiology that is not fully known is whether the observed reduced severity in older children and adults is due to prior exposure to RSV [11, 12], the development of the immune system with age [13–15], or some combination of the two. The epidemiology of RSV throughout the COVID-19 pandemic years changed in many countries [16], primarily due to the use of non-pharmaceutical interventions. These changes, including out-of-season summer peaks and a shift in median age of infection from 8.1 months to 16.4 months observed in Western Australia (WA) [17], has brought the question of RSV severity and its association with age and prior exposure to the forefront. This age shift suggests a higher than usual number of RSV-susceptible children, presumably due to the lack of RSV activity and therefore infection during the winter period, resulting in lower levels of population immunity, otherwise known as an immunity debt. The higher severity in older children, evident by an increase in hospitalisations, is possibly explained by the delay of the first RSV exposure event. Although some increases in viral testing have been seen in observational studies, these increases alone cannot fully explain the out-of-season resurgence and add further weight to the immunity debt hypothesis [17, 18]. Most deterministic compartmental RSV transmission modelling studies published to date have assumed severity wanes with age [14, 19]. Although some studies have modelled prior exposure [20, 21], few have combined age and prior exposure in dynamic models of RSV transmission [22–28]. No models have also incorporated the differential impact of RSV infection in high-risk groups (see [8] for a review of compartmental RSV transmission models up to 2022).

In this study, we present a dynamic compartmental model of RSV transmission fitted to population-based RSV-hospitalisation linked data for 2000–2012 from metropolitan WA, stratified by age and prior exposure. We account for the differential risk of severe RSV disease in full-term and preterm infants. Our main motivation is to provide a foundational model that can be extended to assess the impact of immunisation strategies; including the use of maternal vaccines, monoclonal antibodies administered to infants, or a combination of both, with the potential to evaluate alternative immunisation strategies for high-risk groups including preterm infants. The primary aim of this study is to identify the relationship between age, exposure history, and preterm status to the risk of RSV-related hospitalisation given infection, a key component of RSV models.

## Methods

### Setting and population-based data

WA covers the western third of Australia and has a population of 2.7 million [29]. Approximately 80% of the population resides in the metropolitan region surrounding and including Perth, in the state’s south-west [29]. Data for this study were sourced from a population-based birth cohort study using individually linked administrative health data to investigate the pathogen-specific epidemiology of respiratory infections in children. Full details of the study are reported elsewhere [30]. In brief, data were extracted from the Hospital Morbidity Data Collection, the PathWest Laboratory Database, the Midwives’ Notification System, and the Birth and Death Registry for a whole-of-population cohort of births in WA between 1 January 1996 and 31 December 2012. Data were probabilistically linked using a series of identifiers through the WA Department of Health, with resultant de-identified linked data provided to the research team.

As per our previous analyses of these data [31], PathWest testing records for RSV from respiratory specimens with a specimen collection date between 1 January 2000 and 31 December 2012 were extracted and linked. Thus, RSV detections in this study reflect the pre-COVID-19 pandemic seasonality of RSV that exhibited clear winter peaks (from June-August) in temperate climates of WA (predominantly, the metropolitan region [32]). RSV testing records were merged with hospitalisation records if the date of specimen collection was within 48 h of a hospital admission to reflect the community infection burden of RSV. For this modelling study, we defined an RSV-hospitalisation as any hospital admission where RSV was tested and found positive through immunofluorescence or polymerase chain reaction (PCR). Using information linked through the Midwives’ Notification System, we further separated the RSV-hospitalisations into those children who were born preterm (defined as gestation week at birth less than 37 weeks), or term (defined as gestation week at birth 37 weeks or more). We created a time series of RSV-hospitalisations by preterm and term births between 2000 and 2012 aggregated to a month time period.

### Base and risk model structure and assumptions

**Fig. 1:**
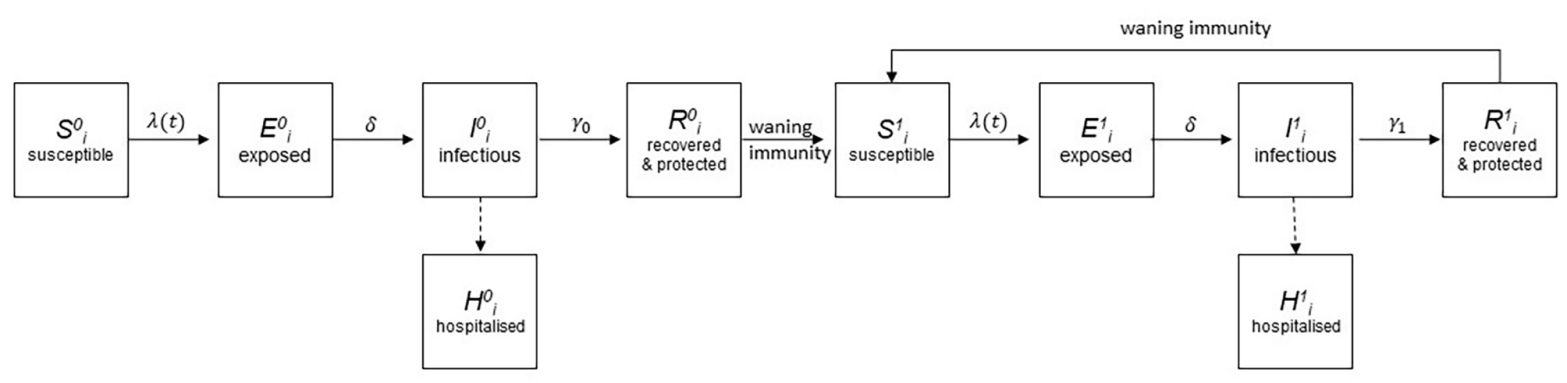
Schematic representation of the RSV transmission base model for each age class i, where each state; susceptible, exposed, infectious, and recovered for both naive (superscript 0) and repeat (superscript 1) exposures, represents a proportion of the total population. 𝝀(t) represents the force of infection, which is driven by the proportion of the population that is infectious and incorporates seasonally-fluctuating transmission

The RSV transmission model is a deterministic compartmental mathematical model of the Susceptible-Exposed-Infectious-Recovered-Susceptible form, as in prior work [14, 15, 19]. We extended this model to capture the progression from first RSV exposure to repeat exposures (Fig. 1). The model divides the population into 75 age groups: 60 one-month groups for individuals younger than 5 years, and five-year age groups thereafter. We assumed a constant population size, based on 2011 Australian Bureau of Statistics (ABS) population data for metropolitan Perth (ages 0–79) [33], with the population distribution over the five-year age groups consistent with ABS data and a uniform population distribution assumed for the monthly age groups. We assumed that the birth and monthly ageing rate was equal to the initial monthly age group population size. In the model, deaths only occur in the oldest age group, with the death and birth rate assumed to be equal. To simulate the monthly ageing between groups we used continuous rather than cohort ageing, where individuals are moved instantaneously at fixed time points to compartments corresponding to older age groups, as used in previous work [14, 15, 19]. We found that continuous ageing, where the ageing rates are included in the ordinary differential equations (ODEs), produced results close to cohort ageing for this model (see Figure S2 in the Supplementary material) and hence were adopted due to simplicity and decrease in computational time. The seasonal nature of RSV transmission was simulated using a cosine function [19] (see Model equations in the Supplementary material). We term this the “base model”.

The “risk model” further divides the population into those born full-term and those born preterm. Using the mean value from our linked dataset of all WA births, the risk model assumes that the proportion of births that are preterm is 0.0849. The risk model replicates the base model structure (see Fig. 1) for preterm and term, allowing a different parameterisation of risk of RSV severity for preterm infants. The equations for the base and risk models are in the Supplementary Material.

### Parameters

Parameterisation of the base and risk compartmental models is summarised in Table 1. We assumed a latent period of four days [34], an infectious period of 10 days for first exposure [35, 36] and 7 days for second and subsequent exposures [35, 36], and that immunity following natural infection lasts for 230 days [19]. As in prior work [15] and aligning with other modelling studies [8], we assumed a reduced susceptibility to RSV infection in the first three months of life due to natural maternal immunity with susceptibility to infection reduced by 92% in the first month of life and 52% in the second and third months. Aligning with other studies [34, 35, 37–42], we assumed a reduction in infectiousness and susceptibility to RSV infection in those who have experienced at least one prior RSV infection. We applied a 30% reduction of infectiousness [35, 37, 38] and a 23% reduction in susceptibility [34, 39–42] as a scaling parameter to individuals that were infected for the second or subsequent time.

A contact matrix representing mixing between age groups in metropolitan Perth was generated using the R package *conmat* [43, 44] to normalise the POLYMOD [45] study contacts (using the United Kingdom data) to the 2011 greater Perth demographic profile (see Figure S3 in the Supplementary Material). As the early age groups are monthly and the contacts in the POLYMOD study are in five-year groups, we made the uniform assumption for the initial five years divided into monthly groups to match the age structure in the model. The daily contact values were converted to monthly values as the model produces monthly numbers of infections and hospitalisations.

**Table 1:** Parameter values for base and risk models

Parameter	Definition	Fixed/Fitted	Value(s)	Reference
1/ɣ_1_	Infectious period (days) for first exposure	Fixed	10	[35, 36]
1/ɣ_2_	Infectious period (days) for second (and subsequent) exposures	Fixed	7	[35, 36]
ῶ	Reduced infectiousness of those who have experienced at least one prior infection	Fixed	0.7	[35, 37, 38]
1/δ	Latent period (days)	Fixed	4	[34]
1/𝜈	Immunity period (days)	Fixed	230	[19]
𝜎_1,_ 𝜎_2,_ 𝜎_3_	Reduced susceptibility due to natural maternal immunity (first, second and third months)	Fixed	𝜎_1_ = 0.08, 𝜎_2_ = 𝜎_3_ = 0.45	[15]
𝜎_E_	Reduced susceptibility due to previous exposure	Fixed	0.77	[34, 39–42]
b_0_	Transmission coefficient	Fitted	0.0204	Fitted value
b_1_	Amplitude of seasonal forcing	Fitted	0.3396	Fitted value
A	Average maximum risk of hospitalisation of term infants (at age 0)	Fitted	0.5144	Fitted value
B	Decay constant	Fitted	0.3776	Fitted value
C	Average minimum risk of hospitalisation across all ages	Fixed	0.015	Fixed value
D	Scaling factor lowering risk for those with a prior exposure	Fixed	0.2	[22, 24]
N	Total population of metropolitan Perth for ages 0–79	Fixed	1,669,809	ABS population 2011 [33]
𝛂	Proportion of births that are preterm (< 37 weeks)	Fixed	0.0849	WA RSV linked data
E	Scaling factor increasing risk for preterm infants	Fitted	2.6329	Fitted value

### Age-to-risk function

In previous work [15], four scaling parameters were used to translate modelled infections to the RSV-hospitalisation time series data for each of four key age groups (0–2 months, 3–5 months, 6–11 months, and 12–23 months). These values, along with parameter values associated with the seasonal forcing function (b_0_ and b_1_, see Table 1), were estimated through a numerical fitting process. To better capture the change in the risk of hospitalisation following infection with age, prior RSV exposure, or preterm risk group, the four parameters were replaced by a simple functional form modelling age-to-risk, with scaling parameters to relate baseline risk in individuals with no prior infection to the (lower) risk in individuals with at least one prior infection, and preterm (higher) risk to baseline term risk. We assumed the age-to-risk function (for the risk model) to take an exponential decay form such that

$$y=(A{e}^{-Bt}+C)DE,$$

where *y* is the modelled relative probabilistic risk of hospitalisation at age *t* (in months), given an individual is infected with RSV, with *A* the average maximum risk increase over the minimum for a term infant (at age 0), B the exponential function decay constant, C the average minimum risk over all ages for all individuals, D a scaling factor (< 1) modifying risk for the prior infection group reflecting a lower risk of hospitalisation, and E a scaling factor (> 1) modifying risk for those born preterm reflecting a higher risk of hospitalisation. We estimate A, B and E by fitting the model to the RSV-hospitalisation time series data. Where E scales the risk to above 1, we assume probability of hospitalisation is 1. The minimum risk parameter C was fixed at 0.015 through exploration of values in the fitting process and the prior infection scaling parameter D was set to 0.2, which is consistent with recent studies [22, 24].

### Model fitting and sensitivity analysis

A Markov-chain Monte Carlo (MCMC) approach using the R package *lazymcmc* [46] was used to fit the model to monthly RSV-hospitalisations for five key age groups (0–2 months, 3–5 months, 6–11 months, 12–23 months and 2 – <5 years) simultaneously. The package uses a Metropolis-Hastings algorithm to sample the posterior multivariate distribution of five parameters in total over a two-step process using a log likelihood based on the assumption that the monthly RSV-hospitalisations follow a Poisson distribution (see Supplementary Material). We first fitted the base model to the time series, estimating two parameters of the seasonal forcing function: b_0_ the transmission coefficient and b_1_ the amplitude of the forcing function, as well as A (the average maximum risk increase for all infants at age 0 months), and B (the constant exponential decay parameter from the age-to-risk function). The forcing function phase shift parameter, ø, was fixed by the assumption that peak infections occurred in July. The posterior samples of model parameters were generated from 8 independent chains of the Metropolis-Hastings sampler, each run for 5000 iterations after an initial, discarded ‘warm-up’ period of 1000 iterations per chain during which the sampler step size was also tuned. Convergence was assessed by visual assessment and diagnostic metrics, ensuring that the potential scale reduction factor for all parameters had values less than 1.1, and that there were at least 1000 effective samples across the 8 chains for each parameter (see Supplementary Material). The estimated values of b_0_, b_1_, A and B resulting from fitting the base model to the time series were then fixed in the risk model before E, the preterm scaling factor, was estimated using the same fitting method as described, with the RSV-hospitalisation time series separated into preterm and term births.

We assessed the sensitivity of the model output to the two fixed parameters in the age-to-risk function, C the average minimum risk of RSV-related hospitalisation for term infants and D the scaling of hospitalisation risk for the prior infection group. As part of the model validation process, we calculated the monthly average age of hospitalisation and compared it to observed data.

## Results

### Model fit

**Fig. 2:**
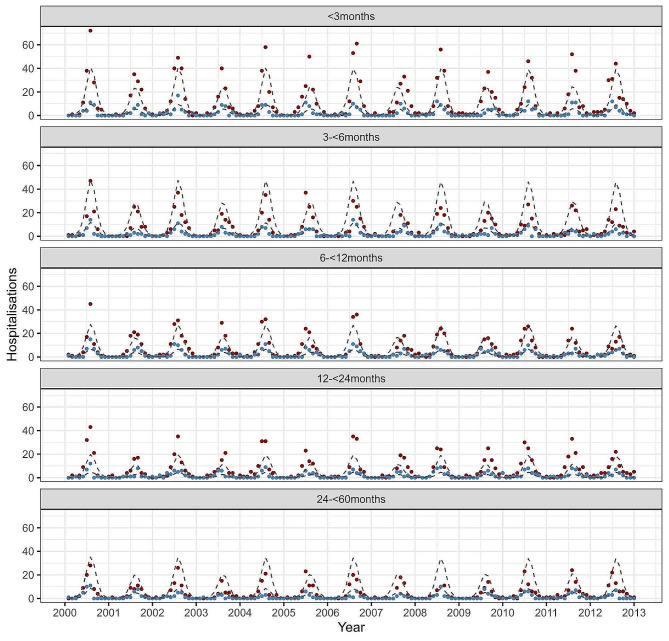
Comparison of the model estimated RSV hospitalisations to the observed time series of the five age groups used to fit the model. The observed hospitalisations are shown with dots (red for term, blue for preterm) and the dashed line is the model output representing estimated hospitalisations. See Figure S4 in Supplementary material for plot comparison of only preterm model estimates to observed data

Figure 2 compares model output to the observed RSV-hospitalisation time series for the five age groups used in fitting (see Supplementary material for model fit diagnostics and plots). All fitted parameter values are given in Table 1, with MCMC distribution point estimates in Table S1 in Supplementary Material. The average age of hospitalisation was calculated for monthly modelled hospitalisations. The overall mean was 13.0 months, with slightly higher average ages in the peak season and lower in off-peak months (see Figure S5 in Supplementary Material). These values are consistent with observed data that shows average age of RSV-hospitalisations during the 3-month peak ranging from 9.8 months to 13.0 months (see Supplementary Material). The model estimates the average age of first RSV infection at 26.1 months (2.2 years), and the average age of second and subsequent infections in children under 5 years old at 41.1 months (3.4 years).

### Age-to-risk fitted parameters

**Fig. 3:**
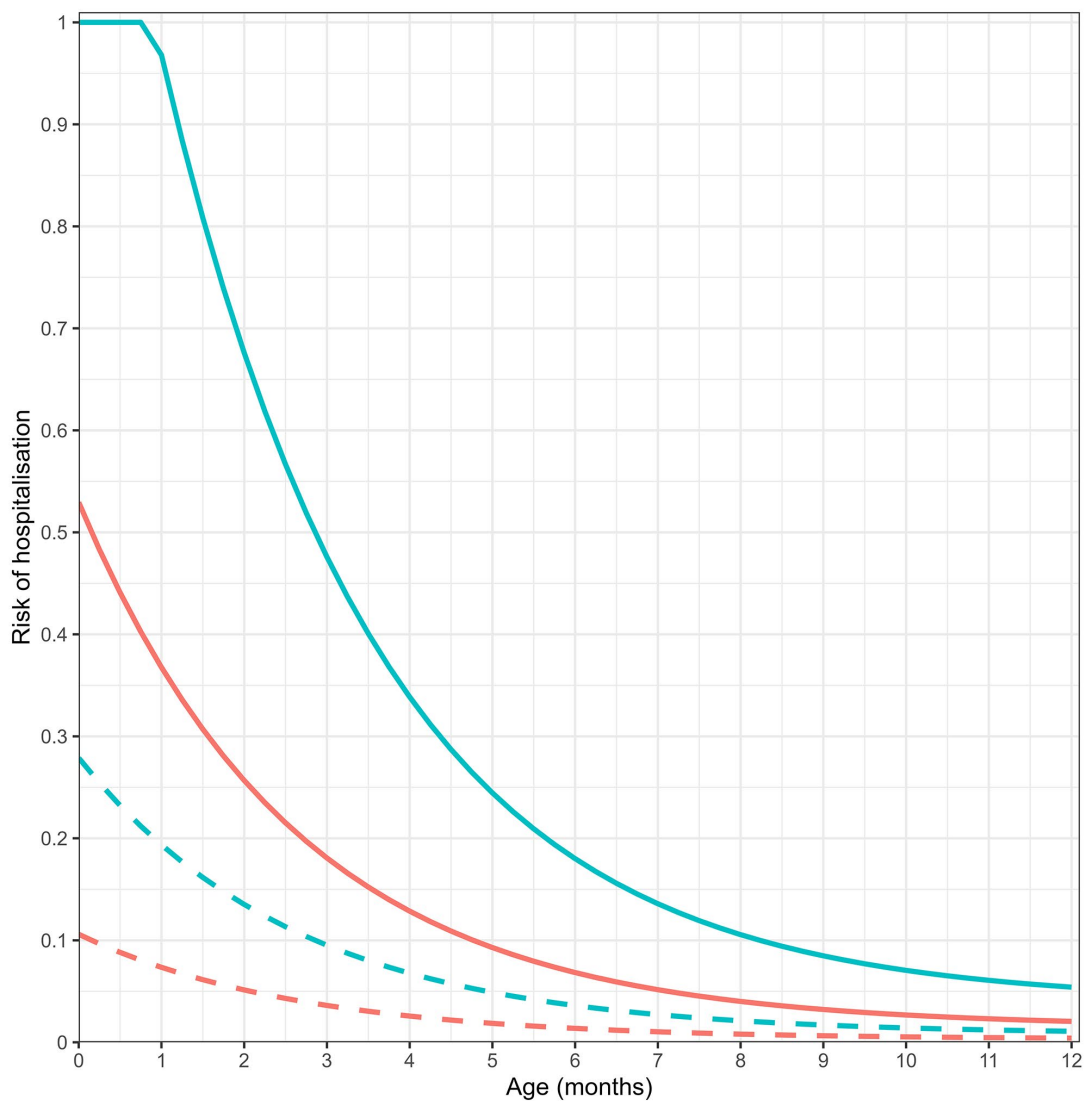
The fitted age-to-risk exponential function relating age (in months) to risk of hospitalisation once infected by RSV. The red curves show risk for those infants born term and blue show the modified risk for preterm birth. Dashed lines relate to the decreased risk for second and subsequent exposures. Fitted values are A = 0.5144, B = 0.3776 and E = 2.6329

Figure 3 shows the age-to-risk function relating age in months to modelled relative risk of hospitalisation in children infected with RSV during the first year of life, parameterised using the fitted values for A, B and E (see Table 1). The fitted function demonstrates that the maximum risk of hospitalisation, given RSV infection, is in preterm infants in their first month of life and that preterm infants are consistently 2.6 times more likely to be hospitalised due to RSV infection than term infants infected at the same age (the fitted value of E). Figure 3 indicates that preterm risk of hospitalisation given infection reaches the equivalent risk of hospitalisation at birth for infants born at term, at approximately 2.5 months of age. The modelled risk of RSV-hospitalisation decays quickly with age in the first year, with term risk decreasing to less than 10% of risk at birth at 7 months of age, and preterm risk decreasing to less than 10% of birth risk at 9 months of age (see Table S2 in Supplementary Material).

### Sensitivity analysis

Sensitivity analysis was conducted with respect to the two fixed parameters of the age-to-risk function. The minimum average risk of hospitalisation for term infants, C, was set to 0.015 in the model. Values of 0.005–0.1 in 0.005 increments were considered in a sensitivity analysis, with all other parameters fixed as in Table 1 and the log likelihoods calculated (see Table S3 in Supplementary Material). The analysis confirmed our default value of C was optimal for the fitted values of transmission and risk function parameters.

The parameter D represents the reduced risk of hospitalisation in those who have experienced a prior RSV infection. Although this scaling effect is represented in Fig. 3, on average this effect will not greatly impact model predictions of hospitalisations as the average age of second and subsequent infections for under 5-year-olds is over 3 years, at which age, risk is already low. The value of D was set to 0.2 to be consistent with previous work [22, 24] but through sensitivity analysis, we explored values between 0 and 1, in increments of 0.05 (see Table S4 in Supplementary Material). We found that the model was not sensitive to the D value, though with a slight preference for low values. As second and subsequent RSV infections for children under 5 years occur when the age-to-risk curve has decayed to close to minimum level risk, there is insufficient data to identify this parameter.

## Conclusions

We developed a dynamic compartmental model of RSV transmission, stratified by age, prior exposure, and preterm status, and fitted to linked laboratory and hospitalisation data for a population cohort in metropolitan Western Australia. The model was fitted using an age-to-risk exponential function that formulates the relationship between age and hospitalisation risk, modified by preterm status and RSV exposure history. This age-to-risk function shows that the risk of hospitalisation given infection declines quickly in the first 12 months of life with risk declining to less than 10% of the risk at birth by age 7 months for term infants.

With both a long-acting monoclonal antibody (mAb) and maternal vaccine now licensed and available in some jurisdictions, and licensure progressing in others, health policymakers need to provide guidance on the most appropriate use of each prevention option. To date, no RSV dynamic transmission model has included stratification by risk group, such as preterm birth [8]. However, our study has demonstrated the importance of including preterm infants in models that will be used to guide pharmaceutical intervention decisions about the administration of a maternal vaccine as an alternative to, or in combination with, a mAb [47]. In our study, we found that for those infants born preterm, the risk of hospitalisation given infection was estimated to be 2.6 times higher than those born at term, with risk decreasing to less than 10% of the risk at birth by the age of 9 months. Infants born preterm are at higher risk of severe RSV infection, most likely due to combinations of an immature cellular innate and adaptive immune system at birth, and smaller airways at birth, leading to increased rates of hospitalisation for acute respiratory infections persisting into early childhood [48]. Infants born preterm are also less likely to be exposed to maternally-derived antibodies. Transplacental transfer of RSV-specific antibodies occurs predominantly in the third trimester of pregnancy [49], the likely time for a maternal vaccine to be administered. For this reason, infants born preterm may benefit more from mAbs than a maternal vaccine. Mathematical models, such as the one developed and fitted in our study, will be important for understanding the implications of the timing of transplacental antibody transfer, and the timing of a mAb and vaccine delivery relative to the RSV season.

Infants born preterm are the largest risk group predisposed to higher rates of severe RSV illness, but other groups include First Nations infants (RSV-hospitalisation rates approximately double the rate of non-First Nations children [50]), those with congenital heart disease, and those with chromosomal abnormalities including Trisomy 21 (ALRI hospitalisation rates are 3–8 times higher than children with no birth defects [51]). Our model structure could be extended to include these other risk groups if sufficient data are available for parameterisation, enabling targeted advice to policymakers for high-risk group specific interventions, for example, the option of an additional dose in a high-risk infant’s second RSV season.

The data used for model fitting are unique to WA due to the availability of longitudinal datasets that can be linked on an individual basis in a total-population setting. These data represent the time period prior to when RSV became a notifiable disease in Australia (from July 2021). Thus, interrogation of routinely collected pathology data from respiratory pathogen testing, as in this study, is the only source of confirmed RSV-hospitalisations. We have previously shown that a combination of laboratory and hospital data are needed to accurately determine RSV infections in children as hospital discharge diagnostic coding alone underestimates the burden of disease [30]. However, despite this strength, our dataset used for model fitting is not without limitations. We did not include laboratory testing at private pathology sites across WA, which is suspected to have increased in recent years, although robust data on the proportion of viral testing in private vs. public laboratories are lacking. Nevertheless, for the years of data used in this study, the level of respiratory viral testing for children conducted outside PathWest as the sole public pathology provider is assumed to be minimal.

Although this model has included the relationship between exposure, age, and severity of RSV infection in the form of the age-to-risk function, a better understanding of the relationship between severity and exposure, in particular, could be gained from further data interrogation that identifies repeat hospitalisations for RSV infections in the same child over a period of time, implying multiple exposures. This is possible with our longitudinal data and currently an area under investigation. Including exposure history in the model could be particularly important when considering the characteristics of immunisation candidates in terms of response after immunity has waned. The COVID-19-era perhaps indicates how immunisation might affect the age profile of hospitalisations, increasing the average age of hospitalisation due to a delay in first exposure [28, 52].

In summary, this work provides an epidemiological model for RSV which can now be adapted, by including additional compartments, to explore the impact of maternal vaccination and the delivery of mAbs, with the preterm sub-population being represented in analyses. We are planning to explore both the individual effects of these interventions as well as combined effects, with consideration of seasonal timing, and identifying optimal immunisation strategies from targeting a risk group such as those born preterm versus a whole-population approach. A key strategy to be explored using our model structure will be a mAb administered to all infants born at the beginning of their first RSV season with a second dose for preterm children in their second year, as was indicated in the Advisory Committee on Immunisation Practices (ACIP) guidelines following the licensure of the new single dose long-acting mAb in the USA [53]. A similar strategy has recently been implemented in Western Australia, the first Australian state to implement and fund a universal mAb program. The first step in model refinement will be to calibrate the model to more recent population-based data from WA, understanding that there may have been a change in RSV dynamics over the intervening time, in particular with a change in population structure and RSV testing behaviour.

### Electronic supplementary material

Below is the link to the electronic supplementary material.

Supplementary Material 1

## Data Availability

The datasets generated and/or analysed during the current study are not publicly available due to the terms of the ethics approval granted by the Western Australian Department of Health Human Research Ethics Committee and data disclosure policies of the Data Providers. The datasets may be available from the corresponding author upon request and subject to approval from the Human Research Ethics Committee and relevant custodians. The R program code is available on github at https://github.com/fionagi/rsvmod.

## Abbreviations

RSV: Respiratory syncytial virus
WA: Western Australia
ALRI: Acute lower respiratory infections
HREC: Human Research Ethics Committee
PCR: Polymerase chain reaction
ABS: Australian Bureau of Statistics
ODEs: Ordinary differential equations
MCMC: Markov-chain Monte Carlo
mAb: Monoclonal antibody
ACIP: Advisory Committee on Immunisation Practices
