# Supplementary material for: Modelling respiratory syncytial virus age-specific risk of hospitalisation in term and preterm infants
Source: BMC Infect Dis. 2024 May 21;24:510. doi: 10.1186/s12879-024-09400-2 (PMC11110433; doi:10.1186/s12879-024-09400-2)
Supplement: Supplementary file 1 — Supplementary Material 1 [file 12879_2024_9400_MOESM1_ESM.docx]

# **Supplementary Material**

## Modelling respiratory syncytial virus age-specific risk of hospitalisation in term and preterm infants

Fiona Giannini, Alexandra B. Hogan, Mohinder Sarna, Kathryn Glass & Hannah C. Moore

## Hospitalisation data for under 5-year-old children


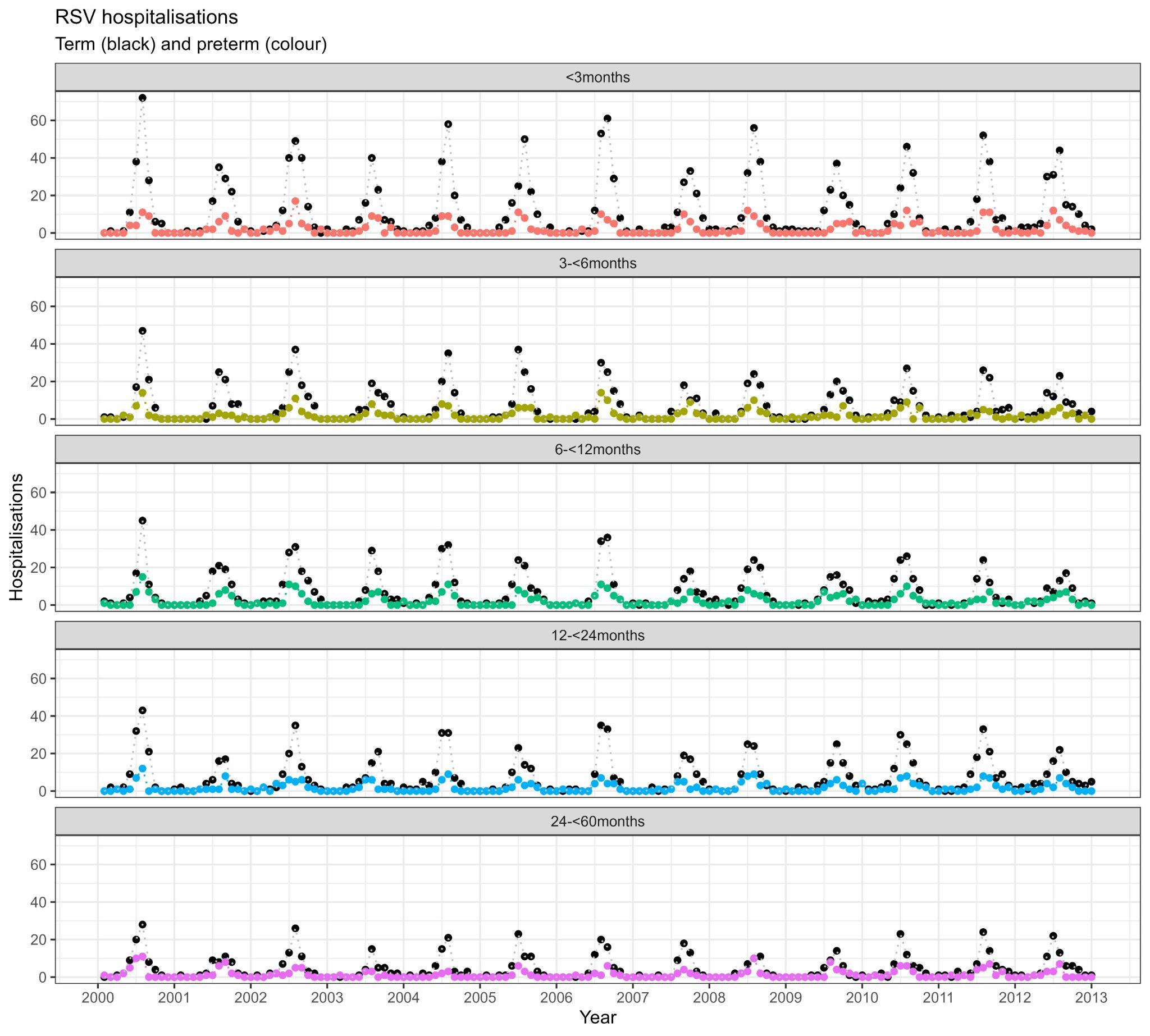


**Figure S1:** Monthly hospitalisations in the Perth metropolitan area for children under 5 years old, divided into infants born term (black dots) and infants born preterm (coloured dots).

## Cohort vs continuous ageing


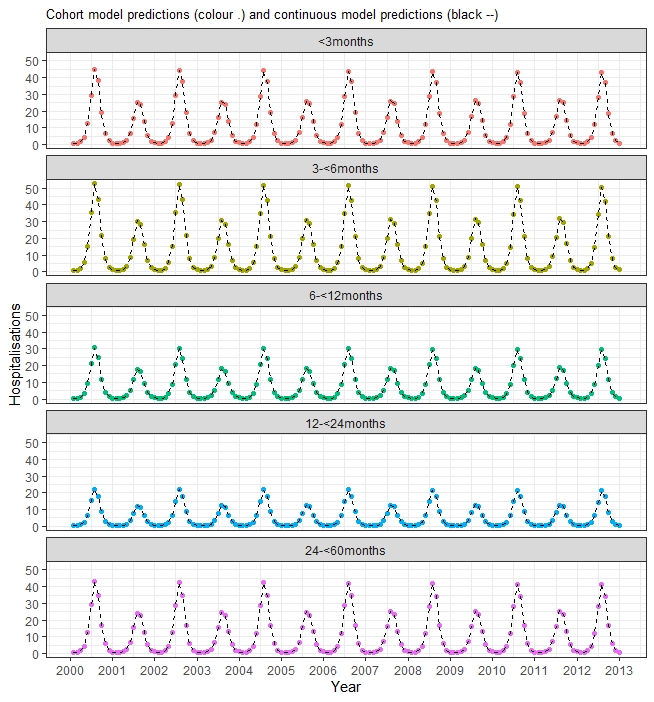


**Figure S2:** Comparison of predicted monthly hospitalisations from the base model with different methods of population ageing. Cohort ageing, where individuals are moved instantaneously at fixed time points to compartments corresponding to the older age groups, is shown in colour, and continuous ageing, where the ageing rates are included in the ordinary differential equations (ODEs), is shown in black. When looking at the absolute difference in number of hospitalisations by age-group and month, there is a median absolute difference of 0.024 hospitalisations per month over the time period, with a maximum absolute difference of 0.547 hospitalisations per month.

## Model equations

The equations below describe the base and risk models respectively, where the risk model further divides the population represented in the base model into those born at term and those born preterm (<37 weeks). In the equations, [
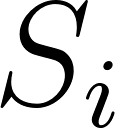
](https://www.codecogs.com/eqnedit.php?latex=S_i#0) represents the number of susceptible individuals in age group [
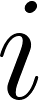
](https://www.codecogs.com/eqnedit.php?latex=i#0), [
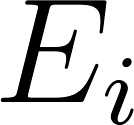
](https://www.codecogs.com/eqnedit.php?latex=E_i#0) represents the number of exposed individuals, [
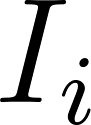
](https://www.codecogs.com/eqnedit.php?latex=I_i#0) represents the number of infectious individuals, and [
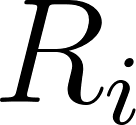
](https://www.codecogs.com/eqnedit.php?latex=R_i#0) represents the number of recovered and temporarily immune individuals. The superscript 0 and 1 indicate naive and subsequent exposures respectively. The indices [
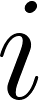
](https://www.codecogs.com/eqnedit.php?latex=i#0) and [
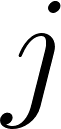
](https://www.codecogs.com/eqnedit.php?latex=j#0) represent the 75 age cohorts where [
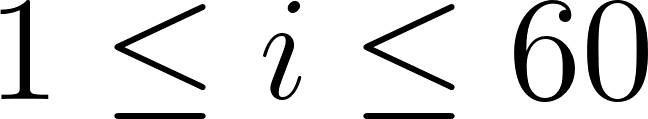
](https://www.codecogs.com/eqnedit.php?latex=1%20%5Cle%20i%20%5Cle%2060#0) are the monthly age groups from 0 to 59 months of age, and then [
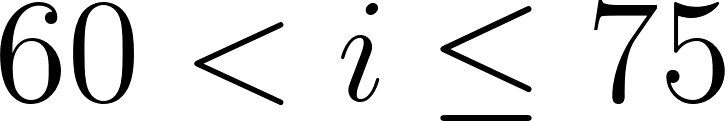
](https://www.codecogs.com/eqnedit.php?latex=60%20%3C%20i%20%5Cle75#0) are 5-year age groups starting at 5-9 years.

The transmission function [
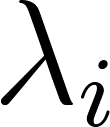
](https://www.codecogs.com/eqnedit.php?latex=%5Clambda_i#0) is the force of infection on age group [
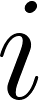
](https://www.codecogs.com/eqnedit.php?latex=i#0) at time [
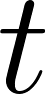
](https://www.codecogs.com/eqnedit.php?latex=t#0). The transmission function phase shift parameter, ø, was fixed by the assumption that peak infections occurred in July, and the contact matrix [
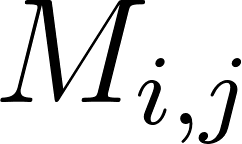
](https://www.codecogs.com/eqnedit.php?latex=M_%7Bi%2Cj%7D#0), represents the number of contacts that an individual in age group [
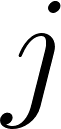
](https://www.codecogs.com/eqnedit.php?latex=j#0) has with individuals in age group [
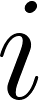
](https://www.codecogs.com/eqnedit.php?latex=i#0). When [
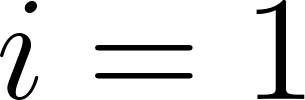
](https://www.codecogs.com/eqnedit.php?latex=i%3D1#0), [
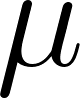
](https://www.codecogs.com/eqnedit.php?latex=%5Cmu#0) is the birth rate and [
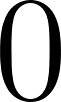
](https://www.codecogs.com/eqnedit.php?latex=0#0) otherwise. The ageing rate of individuals in age group [
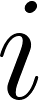
](https://www.codecogs.com/eqnedit.php?latex=i#0) is represented by [
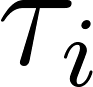
](https://www.codecogs.com/eqnedit.php?latex=%5Ctau_i#0) with [
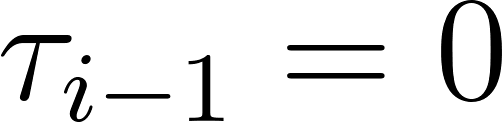
](https://www.codecogs.com/eqnedit.php?latex=%20%5Ctau_%7Bi-1%7D%20%3D%200#0) if [
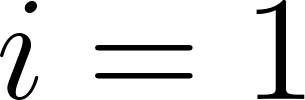
](https://www.codecogs.com/eqnedit.php?latex=i%3D1#0) and [
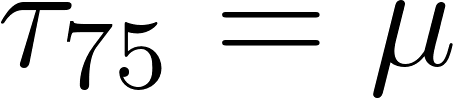
](https://www.codecogs.com/eqnedit.php?latex=%20%5Ctau_%7B75%7D%20%3D%5Cmu#0) as mortality only occurs in the oldest age group and is equal to the birth rate in the closed system. The scaling of susceptibility dependent on age (including susceptibility reduction due to natural immunity in the first three months) is represented by [
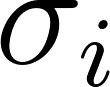
](https://www.codecogs.com/eqnedit.php?latex=%5Csigma_i#0) and [
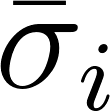
](https://www.codecogs.com/eqnedit.php?latex=%5Cbar%7B%5Csigma%7D_i#0) is the combination of scaling susceptibility due to age and prior exposure. All other parameters are defined in Table 1.

*Base model*

The base model can be represented by the following equations,

[
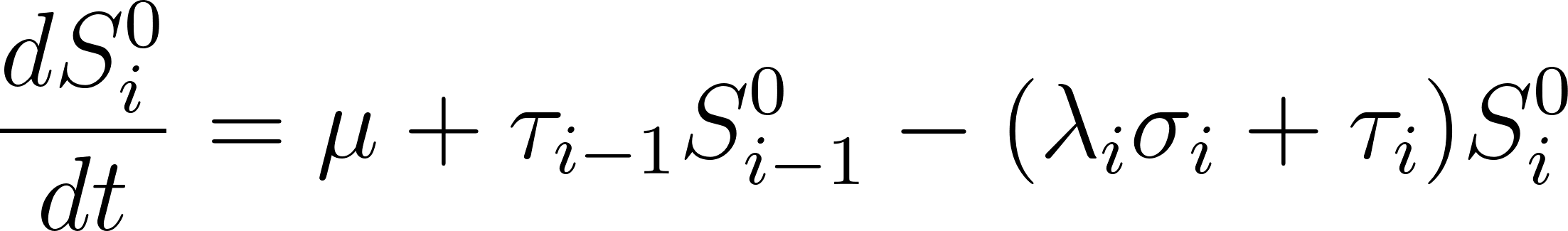
](https://www.codecogs.com/eqnedit.php?latex=%5Cfrac%7Bd%7BS%5E0_i%7D%7D%7Bdt%7D%20%3D%20%5Cmu%20%2B%20%5Ctau_%7Bi-1%7D%7BS%5E0_%7Bi-1%7D%7D%20-%20(%5Clambda_i%20%5Csigma_i%20%2B%20%5Ctau_i)S%5E0_i#0)

( 1 )

[
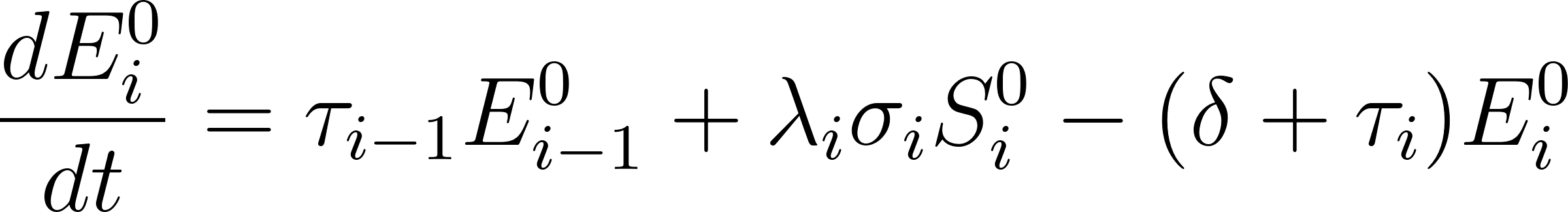
](https://www.codecogs.com/eqnedit.php?latex=%20%5Cfrac%7BdE%5E0_i%7D%7Bdt%7D%20%3D%20%5Ctau_%7Bi-1%7DE%5E0_%7Bi-1%7D%2B%5Clambda_i%20%5Csigma_i%20S%5E0_i%20-%20(%5Cdelta%20%2B%20%5Ctau_i)%20E%5E0_i#0)

( 2 )

[
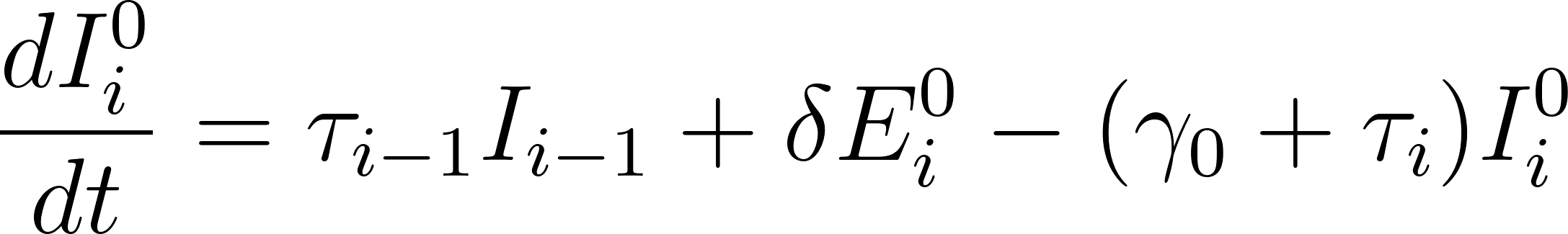
](https://www.codecogs.com/eqnedit.php?latex=%5Cfrac%7BdI%5E0_i%7D%7Bdt%7D%20%3D%20%5Ctau_%7Bi-1%7DI_%7Bi-1%7D%2B%5Cdelta%20E%5E0_i%20-%20(%5Cgamma_0%20%2B%20%5Ctau_i)%20I%5E0_i#0)

( 3 )

[
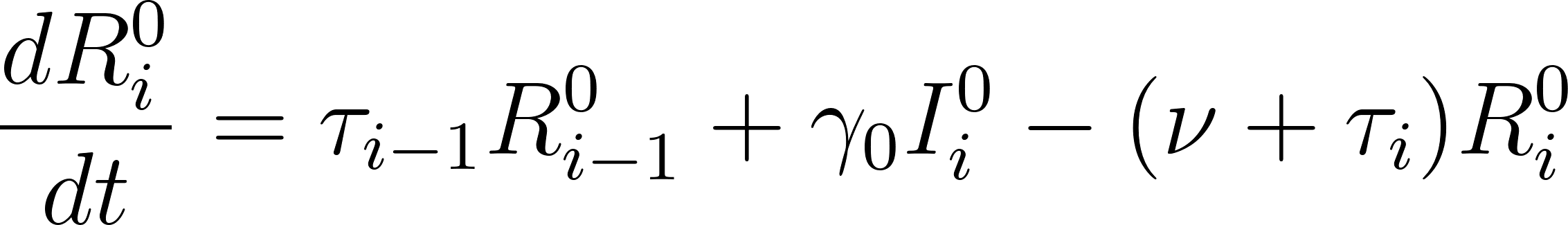
](https://www.codecogs.com/eqnedit.php?latex=%5Cfrac%7BdR%5E0_i%7D%7Bdt%7D%20%3D%20%5Ctau_%7Bi-1%7DR%5E0_%7Bi-1%7D%2B%5Cgamma_0%20I%5E0_i%20-%20(%5Cnu%20%2B%20%5Ctau_i)%20R%5E0_i#0)

( 4 )

[
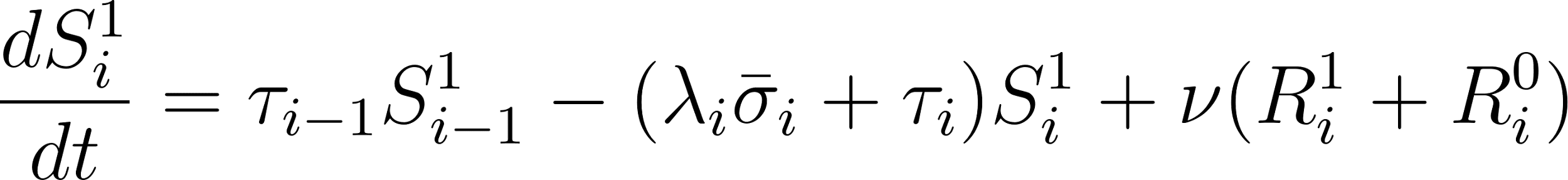
](https://www.codecogs.com/eqnedit.php?latex=%5Cfrac%7BdS%5E1_i%7D%7Bdt%7D%20%3D%20%5Ctau_%7Bi-1%7DS%5E1_%7Bi-1%7D%20-%20(%5Clambda_i%20%5Cbar%7B%5Csigma%7D_i%20%2B%20%5Ctau_i)S%5E1_i%20%2B%20%5Cnu%20(R%5E1_i%20%2B%20R%5E0_i)#0)

( 5 )

[
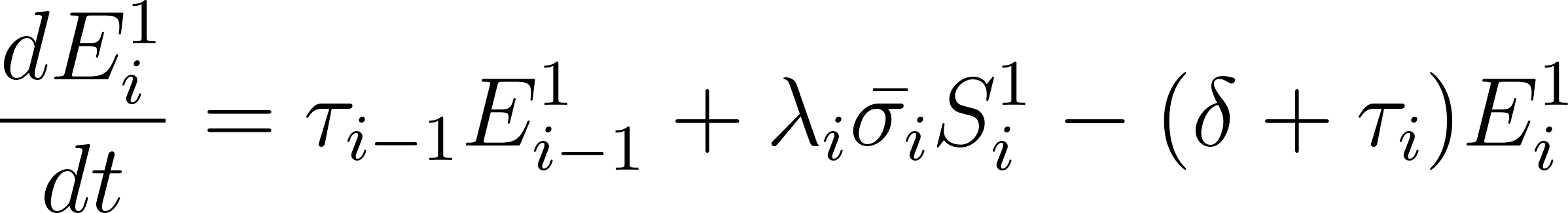
](https://www.codecogs.com/eqnedit.php?latex=%20%5Cfrac%7BdE%5E1_i%7D%7Bdt%7D%20%3D%20%5Ctau_%7Bi-1%7DE%5E1_%7Bi-1%7D%2B%5Clambda_i%20%5Cbar%7B%5Csigma_i%7D%20S%5E1_i%20-%20(%5Cdelta%20%2B%20%5Ctau_i)%20E%5E1_i#0)

( 6 )

[
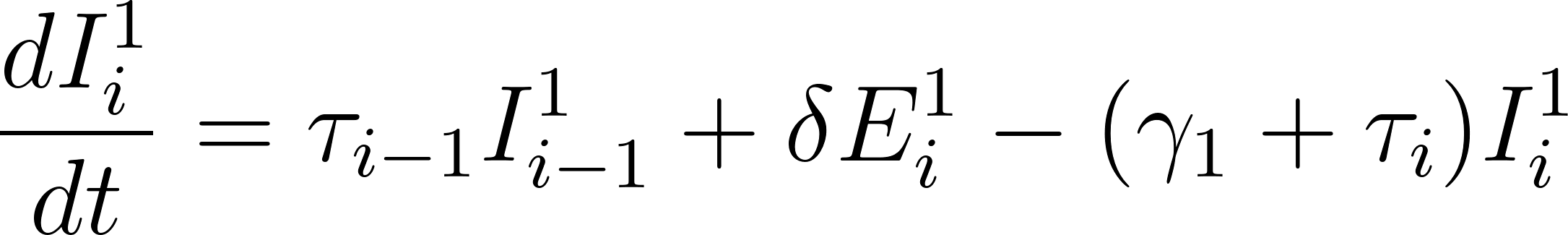
](https://www.codecogs.com/eqnedit.php?latex=%5Cfrac%7BdI%5E1_i%7D%7Bdt%7D%20%3D%20%5Ctau_%7Bi-1%7DI%5E1_%7Bi-1%7D%2B%5Cdelta%20E%5E1_i%20-%20(%5Cgamma_1%20%2B%20%5Ctau_i)%20I%5E1_i#0)

( 7 )

[
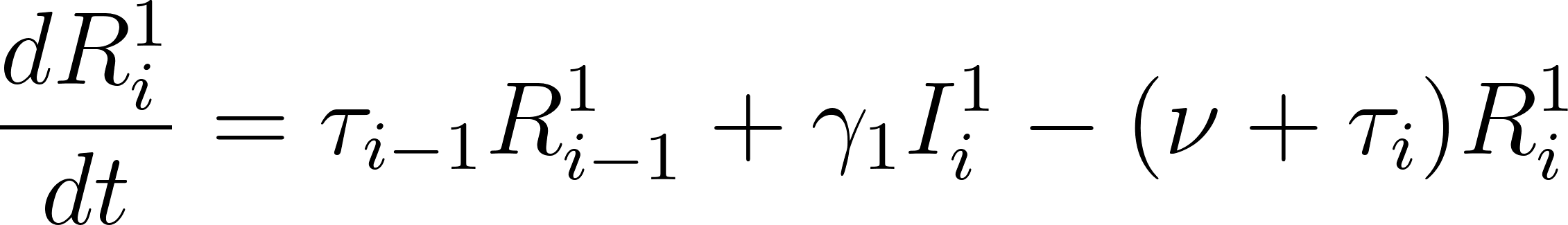
](https://www.codecogs.com/eqnedit.php?latex=%5Cfrac%7BdR%5E1_i%7D%7Bdt%7D%20%3D%20%5Ctau_%7Bi-1%7DR%5E1_%7Bi-1%7D%2B%5Cgamma_1%20I%5E1_i%20-%20(%5Cnu%20%2B%20%5Ctau_i)%20R%5E1_i#0)

( 8 )

The transmission function is represented by the following,

[[
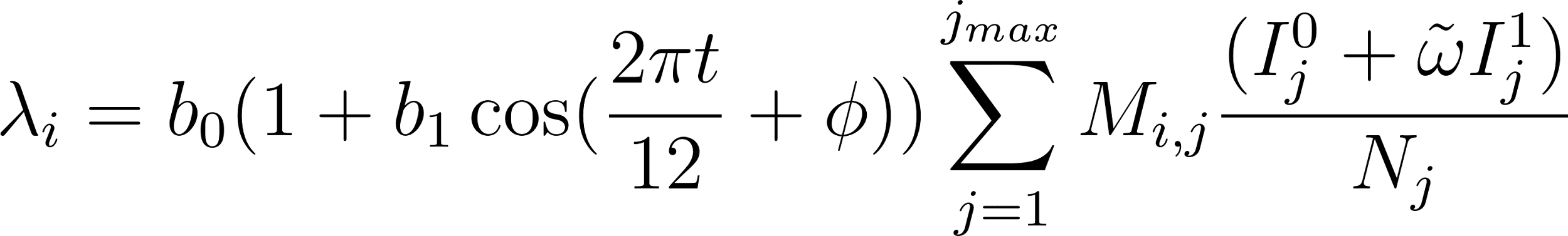
](https://www.codecogs.com/eqnedit.php?latex=%5Clambda_i%20%3D%20b_0(1%2Bb_1%5Ccos%20(%5Cfrac%7B2%5Cpi%20t%7D%7B12%7D%2B%5Cphi))%5Csum_%7Bj%3D1%7D%5E%7Bj_%7Bmax%7D%7DM_%7Bi%2Cj%7D%20%5Cfrac%7B(I%5E0_j%2B%5Ctilde%7B%5Comega%7DI%5E1_j)%7D%7BN_j%7D#0)](https://www.codecogs.com/eqnedit.php?latex=%5Clambda_i%20%3D%20b_0(1%2Bb_1%5Ccos%20(%5Cfrac%7B2%5Cpi%20t%7D%7B12%7D%2B%5Cphi))%5Cfrac%7B1%7D%7BN_i%7D%5Csum_%7Bj%3D1%7D%5E%7Bj_%7Bmax%7D%7DM_%7Bi%2Cj%7D%20(I%5E0_j%2B%5Ctilde%7B%5Comega%7DI%5E1_j)#0)

( 9 )

*Risk model*

The risk model can be represented by the following equations, noting the equations using variables denoted with a bar correspond to the pre-term risk group and without the bar, the term population,

[
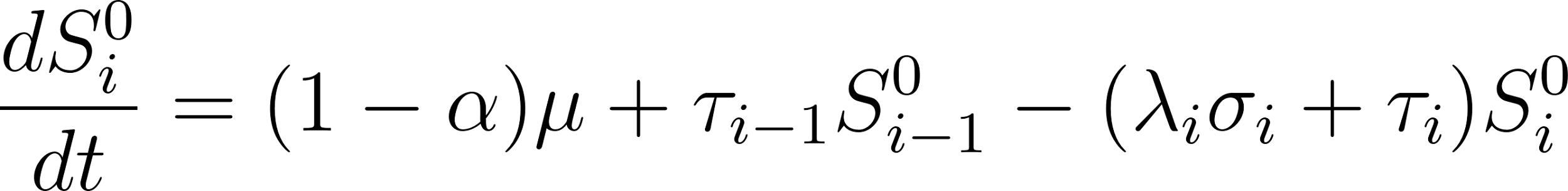
](https://www.codecogs.com/eqnedit.php?latex=%5Cfrac%7Bd%7BS%5E0_i%7D%7D%7Bdt%7D%20%3D%20(1-%20%5Calpha)%5Cmu%20%2B%20%5Ctau_%7Bi-1%7D%7BS%5E0_%7Bi-1%7D%7D%20-%20(%5Clambda_i%20%5Csigma_i%20%2B%20%5Ctau_i)S%5E0_i#0)(population born at term)

( 10 )

[
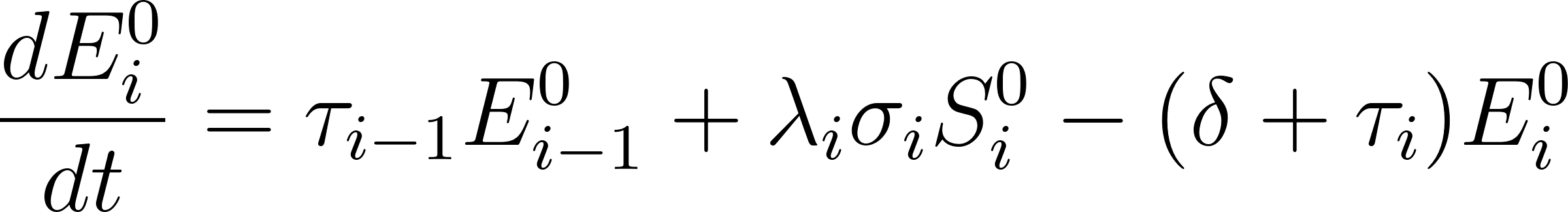
](https://www.codecogs.com/eqnedit.php?latex=%20%5Cfrac%7BdE%5E0_i%7D%7Bdt%7D%20%3D%20%5Ctau_%7Bi-1%7DE%5E0_%7Bi-1%7D%2B%5Clambda_i%20%5Csigma_i%20S%5E0_i%20-%20(%5Cdelta%20%2B%20%5Ctau_i)%20E%5E0_i#0)

( 11 )

[
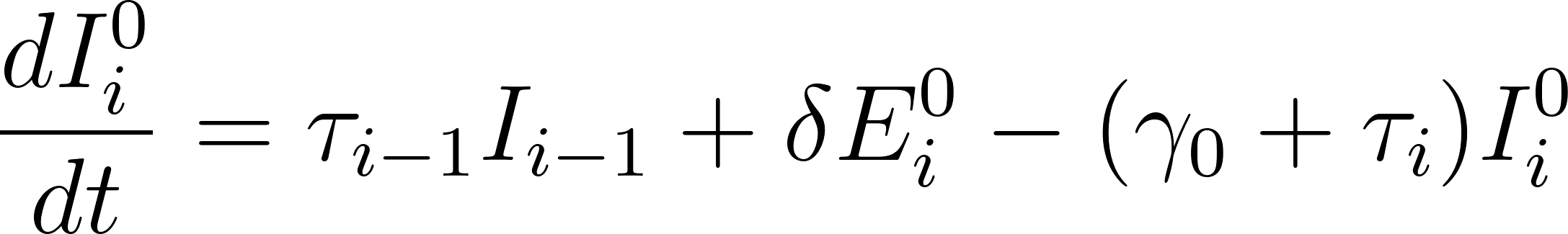
](https://www.codecogs.com/eqnedit.php?latex=%5Cfrac%7BdI%5E0_i%7D%7Bdt%7D%20%3D%20%5Ctau_%7Bi-1%7DI_%7Bi-1%7D%2B%5Cdelta%20E%5E0_i%20-%20(%5Cgamma_0%20%2B%20%5Ctau_i)%20I%5E0_i#0)

( 12 )

[
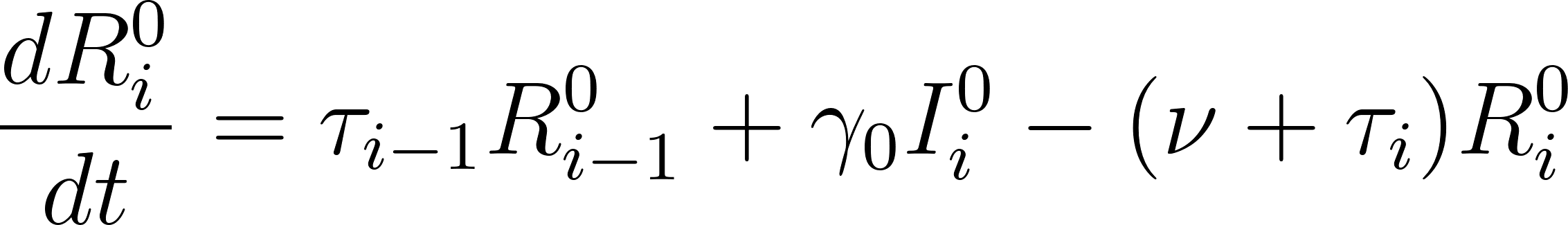
](https://www.codecogs.com/eqnedit.php?latex=%5Cfrac%7BdR%5E0_i%7D%7Bdt%7D%20%3D%20%5Ctau_%7Bi-1%7DR%5E0_%7Bi-1%7D%2B%5Cgamma_0%20I%5E0_i%20-%20(%5Cnu%20%2B%20%5Ctau_i)%20R%5E0_i#0)

( 13 )

[
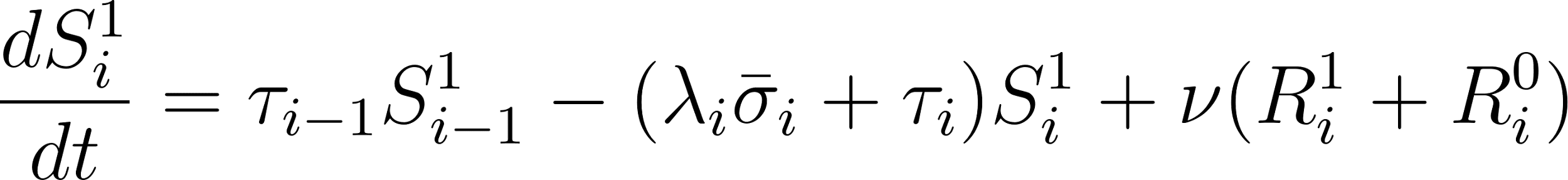
](https://www.codecogs.com/eqnedit.php?latex=%5Cfrac%7BdS%5E1_i%7D%7Bdt%7D%20%3D%20%5Ctau_%7Bi-1%7DS%5E1_%7Bi-1%7D%20-%20(%5Clambda_i%20%5Cbar%7B%5Csigma%7D_i%20%2B%20%5Ctau_i)S%5E1_i%20%2B%20%5Cnu%20(R%5E1_i%20%2B%20R%5E0_i)#0)

( 14 )

[
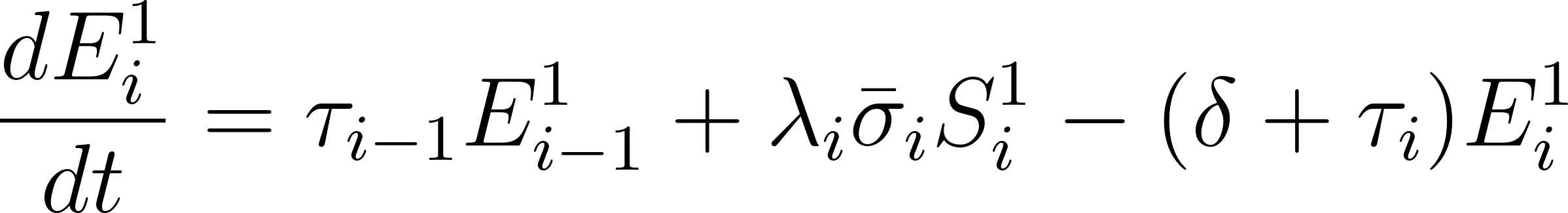
](https://www.codecogs.com/eqnedit.php?latex=%20%5Cfrac%7BdE%5E1_i%7D%7Bdt%7D%20%3D%20%5Ctau_%7Bi-1%7DE%5E1_%7Bi-1%7D%2B%5Clambda_i%20%5Cbar%7B%5Csigma%7D_i%20S%5E1_i%20-%20(%5Cdelta%20%2B%20%5Ctau_i)%20E%5E1_i#0)

( 15 )

[
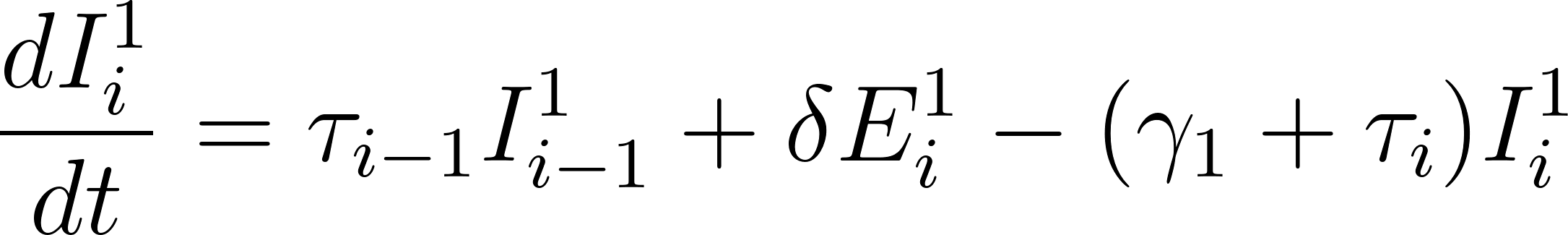
](https://www.codecogs.com/eqnedit.php?latex=%5Cfrac%7BdI%5E1_i%7D%7Bdt%7D%20%3D%20%5Ctau_%7Bi-1%7DI%5E1_%7Bi-1%7D%2B%5Cdelta%20E%5E1_i%20-%20(%5Cgamma_1%20%2B%20%5Ctau_i)%20I%5E1_i#0)

( 16 )

[
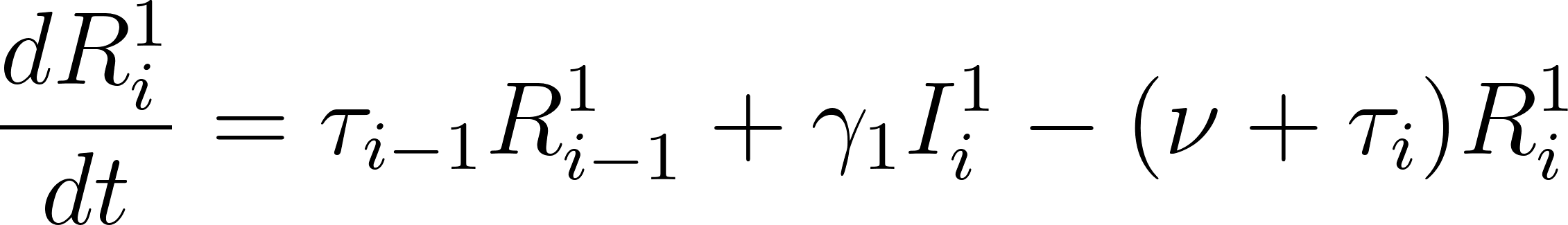
](https://www.codecogs.com/eqnedit.php?latex=%5Cfrac%7BdR%5E1_i%7D%7Bdt%7D%20%3D%20%5Ctau_%7Bi-1%7DR%5E1_%7Bi-1%7D%2B%5Cgamma_1%20I%5E1_i%20-%20(%5Cnu%20%2B%20%5Ctau_i)%20R%5E1_i#0)

( 17 )

[
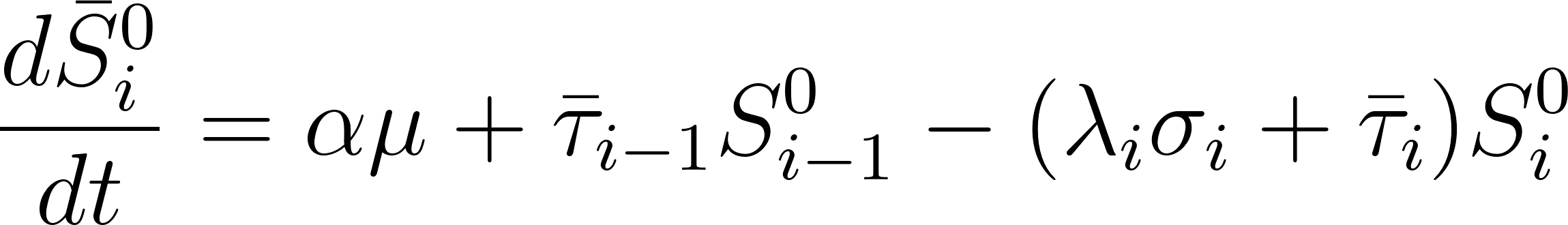
](https://www.codecogs.com/eqnedit.php?latex=%5Cfrac%7Bd%7B%5Cbar%7BS%7D%5E0_i%7D%7D%7Bdt%7D%20%3D%20%5Calpha%5Cmu%20%2B%20%5Cbar%7B%5Ctau%7D_%7Bi-1%7D%7BS%5E0_%7Bi-1%7D%7D%20-%20(%5Clambda_i%20%5Csigma_i%20%2B%20%5Cbar%7B%5Ctau%7D_i)S%5E0_i#0) (population born preterm)

( 18 )

[
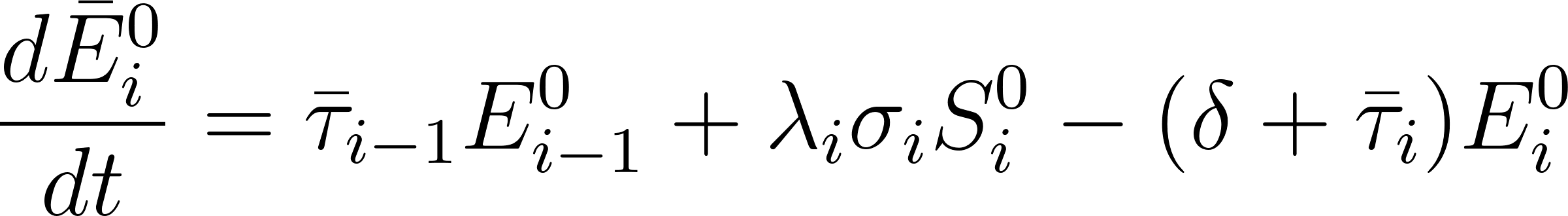
](https://www.codecogs.com/eqnedit.php?latex=%20%5Cfrac%7Bd%5Cbar%7BE%7D%5E0_i%7D%7Bdt%7D%20%3D%20%5Cbar%7B%5Ctau%7D_%7Bi-1%7DE%5E0_%7Bi-1%7D%2B%5Clambda_i%20%5Csigma_i%20S%5E0_i%20-%20(%5Cdelta%20%2B%20%20%5Cbar%7B%5Ctau%7D_i)%20E%5E0_i#0)

( 19 )

[
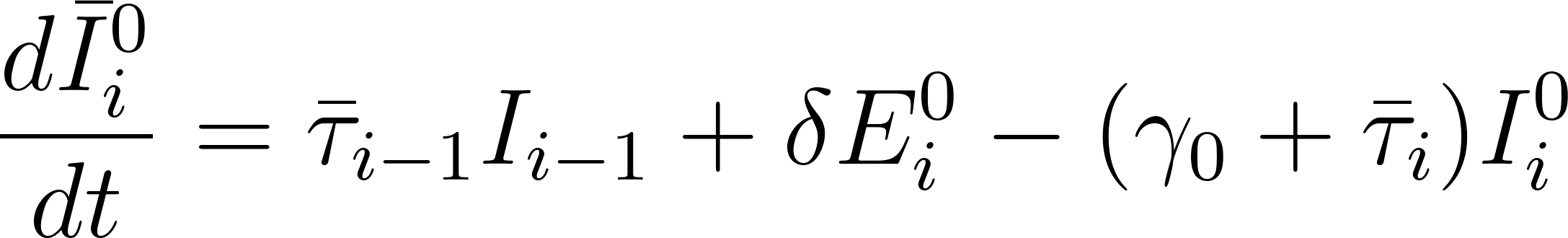
](https://www.codecogs.com/eqnedit.php?latex=%5Cfrac%7Bd%5Cbar%7BI%7D%5E0_i%7D%7Bdt%7D%20%3D%20%20%5Cbar%7B%5Ctau%7D_%7Bi-1%7DI_%7Bi-1%7D%2B%5Cdelta%20E%5E0_i%20-%20(%5Cgamma_0%20%2B%20%20%5Cbar%7B%5Ctau%7D_i)%20I%5E0_i#0)

( 20 )

[
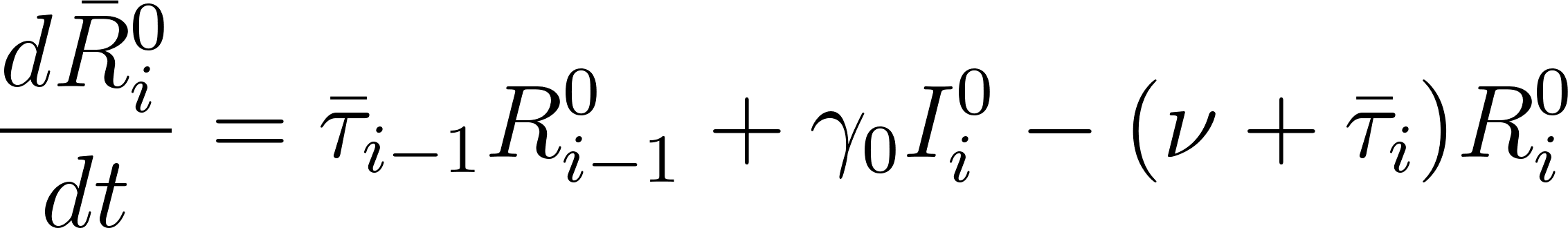
](https://www.codecogs.com/eqnedit.php?latex=%5Cfrac%7Bd%5Cbar%7BR%7D%5E0_i%7D%7Bdt%7D%20%3D%20%20%5Cbar%7B%5Ctau%7D_%7Bi-1%7DR%5E0_%7Bi-1%7D%2B%5Cgamma_0%20I%5E0_i%20-%20(%5Cnu%20%2B%20%20%5Cbar%7B%5Ctau%7D_i)%20R%5E0_i#0)

( 21 )

[
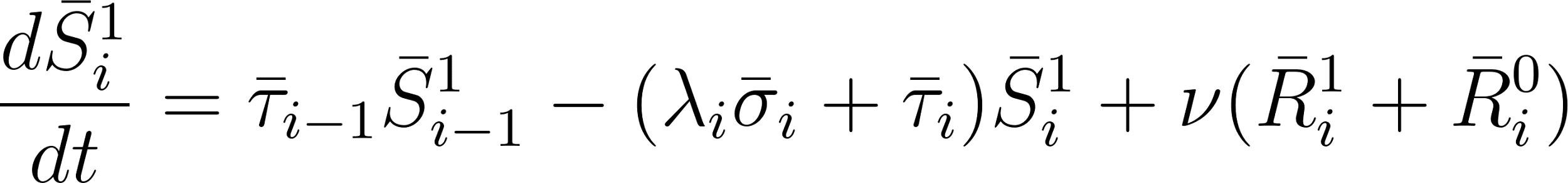
](https://www.codecogs.com/eqnedit.php?latex=%5Cfrac%7Bd%5Cbar%7BS%7D%5E1_i%7D%7Bdt%7D%20%3D%20%20%5Cbar%7B%5Ctau%7D_%7Bi-1%7D%5Cbar%7BS%7D%5E1_%7Bi-1%7D%20-%20(%5Clambda_i%20%5Cbar%7B%5Csigma%7D_i%20%2B%20%20%5Cbar%7B%5Ctau%7D_i)%5Cbar%7BS%7D%5E1_i%20%2B%20%5Cnu%20(%5Cbar%7BR%7D%5E1_i%20%2B%20%5Cbar%7BR%7D%5E0_i)#0)

( 22 )

[
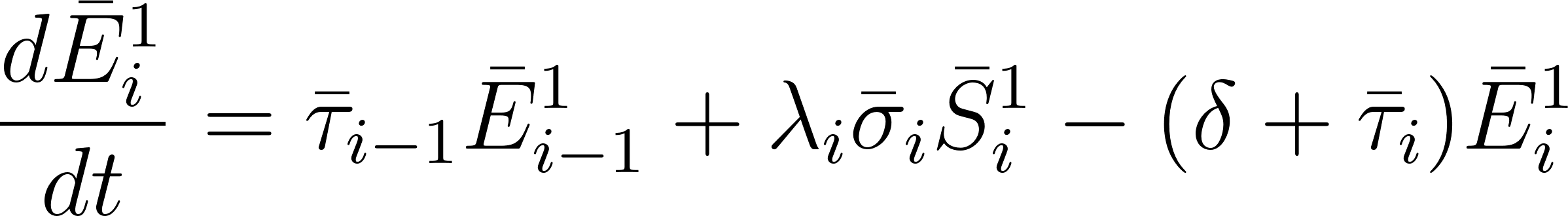
](https://www.codecogs.com/eqnedit.php?latex=%20%5Cfrac%7Bd%5Cbar%7BE%7D%5E1_i%7D%7Bdt%7D%20%3D%20%20%5Cbar%7B%5Ctau%7D_%7Bi-1%7D%5Cbar%7BE%7D%5E1_%7Bi-1%7D%2B%5Clambda_i%20%5Cbar%7B%5Csigma%7D_i%20%5Cbar%7BS%7D%5E1_i%20-%20(%5Cdelta%20%2B%20%20%5Cbar%7B%5Ctau%7D_i)%20%5Cbar%7BE%7D%5E1_i#0)

( 23 )

[
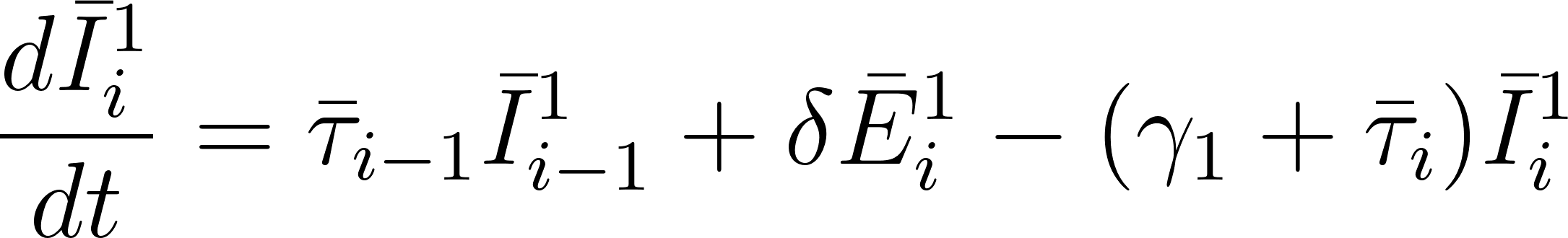
](https://www.codecogs.com/eqnedit.php?latex=%5Cfrac%7Bd%5Cbar%7BI%7D%5E1_i%7D%7Bdt%7D%20%3D%20%20%5Cbar%7B%5Ctau%7D_%7Bi-1%7D%5Cbar%7BI%7D%5E1_%7Bi-1%7D%2B%5Cdelta%20%5Cbar%7BE%7D%5E1_i%20-%20(%5Cgamma_1%20%2B%20%20%5Cbar%7B%5Ctau%7D_i)%20%5Cbar%7BI%7D%5E1_i#0)

( 24 )

[
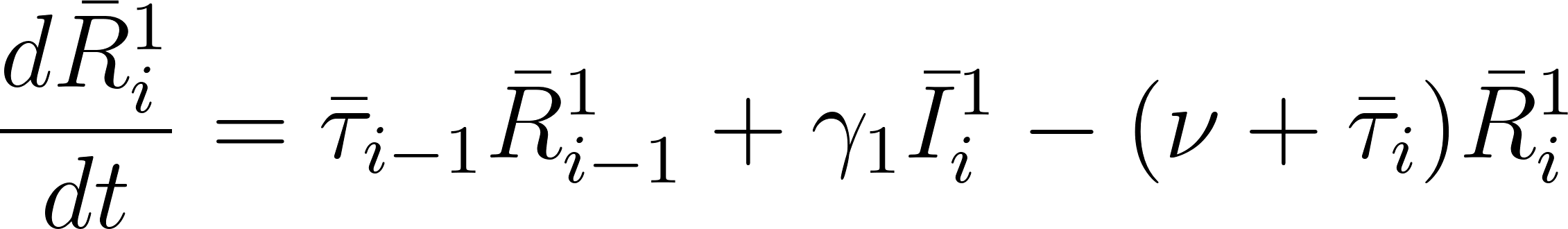
](https://www.codecogs.com/eqnedit.php?latex=%5Cfrac%7Bd%5Cbar%7BR%7D%5E1_i%7D%7Bdt%7D%20%3D%20%20%5Cbar%7B%5Ctau%7D_%7Bi-1%7D%5Cbar%7BR%7D%5E1_%7Bi-1%7D%2B%5Cgamma_1%20%5Cbar%7BI%7D%5E1_i%20-%20(%5Cnu%20%2B%20%20%5Cbar%7B%5Ctau%7D_i)%20%5Cbar%7BR%7D%5E1_i#0)

( 25 )

The transmission function is represented by the following,

[
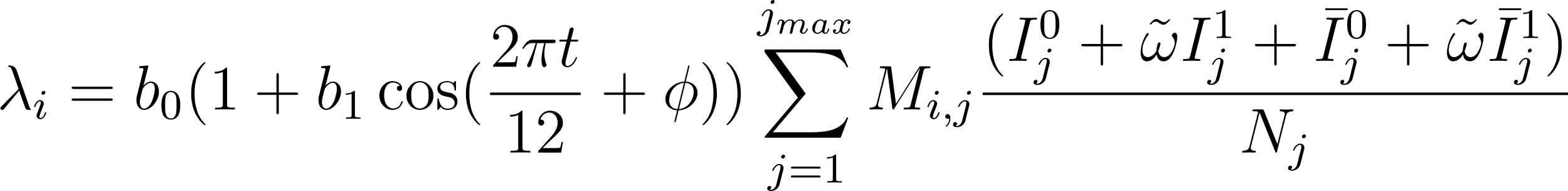
](https://www.codecogs.com/eqnedit.php?latex=%5Clambda_i%20%3D%20b_0(1%2Bb_1%5Ccos%20(%5Cfrac%7B2%5Cpi%20t%7D%7B12%7D%2B%5Cphi))%5Csum_%7Bj%3D1%7D%5E%7Bj_%7Bmax%7D%7DM_%7Bi%2Cj%7D%5Cfrac%7B(I%5E0_j%2B%5Ctilde%7B%5Comega%7DI%5E1_j%2B%20%5Cbar%7BI%7D%5E0_j%2B%5Ctilde%7B%5Comega%7D%5Cbar%7BI%7D%5E1_j)%7D%7BN_j%7D#0)

( 26 )

## Contact matrix


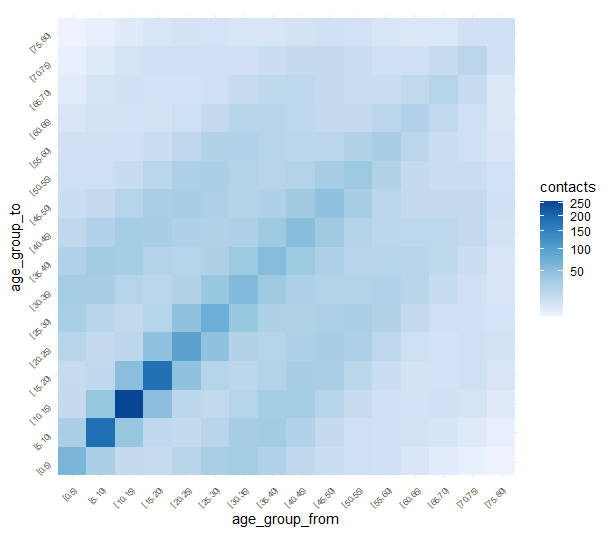


**Figure S3:** Monthly number of contacts between five-year age groups within the population. This contact matrix was generated using the R package *conmat* based on the POLYMOD study results for the UK, normalised to 2011 metropolitan Perth population demographics. The [0,5) year age group is divided into monthly age groups in the model, with contacts being uniformly distributed into these resulting age groups.

## Model fitting

### Log likelihood

We fitted the parameters b_0,_ b_1,_ A, B and E to RSV-hospitalisation data using maximum likelihood estimation. To derive the log likelihood, we assumed that the number of hospitalisations each month followed a Poisson distribution. For the risk model, the log likelihood, [
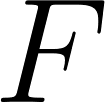
](https://www.codecogs.com/eqnedit.php?latex=F#0) is the sum of the log likelihood of the term, [
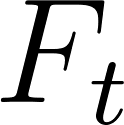
](https://www.codecogs.com/eqnedit.php?latex=F_t#0), and preterm, [
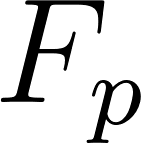
](https://www.codecogs.com/eqnedit.php?latex=F_p#0), components of the model,

[
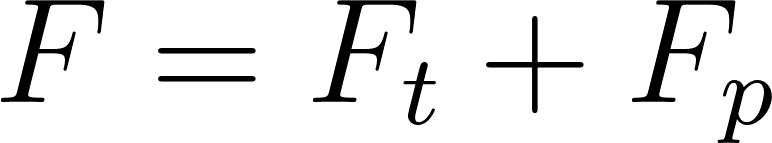
](https://www.codecogs.com/eqnedit.php?latex=F%20%3D%20F_t%20%2B%20F_p#0)

with

[
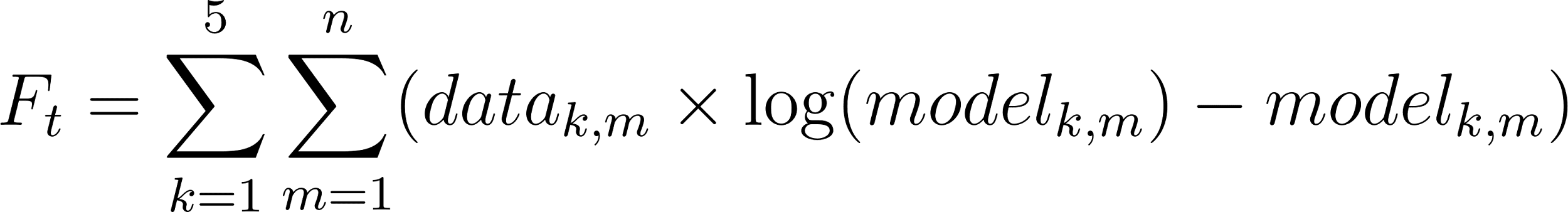
](https://www.codecogs.com/eqnedit.php?latex=F_t%20%3D%20%5Csum_%7Bk%3D1%7D%5E%7B5%7D%5Csum_%7Bm%3D1%7D%5E%7Bn%7D(data_%7Bk%2Cm%7D%20%5Ctimes%20%5Clog(model_%7Bk%2Cm%7D)%20-%20model_%7Bk%2Cm%7D)#0)

and

[
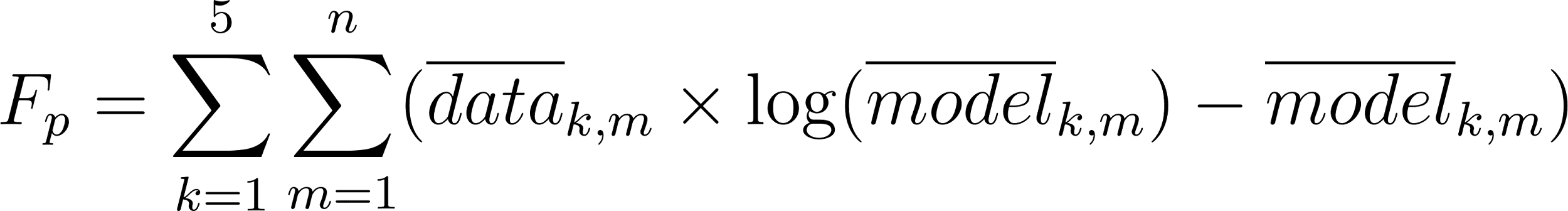
](https://www.codecogs.com/eqnedit.php?latex=F_p%20%3D%20%5Csum_%7Bk%3D1%7D%5E%7B5%7D%5Csum_%7Bm%3D1%7D%5E%7Bn%7D(%5Coverline%7Bdata%7D_%7Bk%2Cm%7D%20%5Ctimes%20%5Clog(%5Coverline%7Bmodel%7D_%7Bk%2Cm%7D)%20-%20%5Coverline%7Bmodel%7D_%7Bk%2Cm%7D)#0)

( 27 )

where [
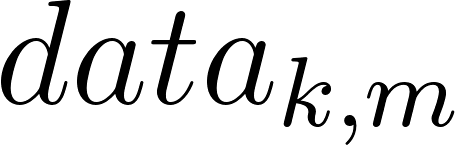
](https://www.codecogs.com/eqnedit.php?latex=data_%7Bk%2Cm%7D#0) and [
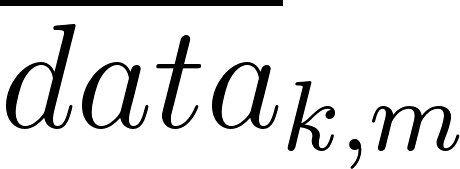
](https://www.codecogs.com/eqnedit.php?latex=%5Coverline%7Bdata%7D_%7Bk%2Cm%7D#0) are the numbers of term and preterm RSV hospitalisations respectively of children in age-group [
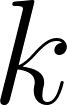
](https://www.codecogs.com/eqnedit.php?latex=k#0) and month [
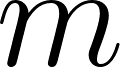
](https://www.codecogs.com/eqnedit.php?latex=m#0), and [
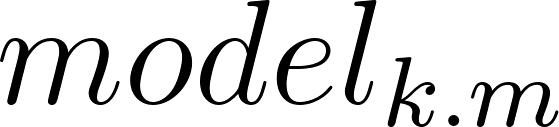
](https://www.codecogs.com/eqnedit.php?latex=model_%7Bk.m%7D#0) and [
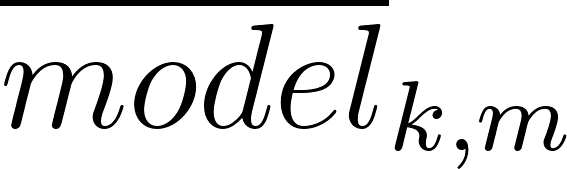
](https://www.codecogs.com/eqnedit.php?latex=%5Coverline%7Bmodel%7D_%7Bk%2Cm%7D#0) are the model predicted term and preterm RSV hospitalisation of children in age-group [
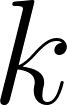
](https://www.codecogs.com/eqnedit.php?latex=k#0) and month [
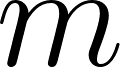
](https://www.codecogs.com/eqnedit.php?latex=m#0). The subscript [
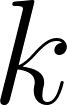
](https://www.codecogs.com/eqnedit.php?latex=k#0) denotes the five age-groups: 0-2 months, 3-5 months, 6-11 months, 12-23 months and 2-<5 years.

### Parameter estimates

**Table S1:** Estimated maximum likelihood values for b_0_ the transmission coefficient, b_1_ the amplitude of the forcing function, A the average maximum risk increase for all infants at age 0 months and B the constant exponential decay parameter from the age-to-risk function. The Effective Sample Size (ESS) is the number of effectively independent draws from the posterior distribution that the Markov chain is equivalent to. The mean and 2.5, 25, 50, 75, and 97.5 percentile estimates are also provided.

| Parameter | ESS | Maximum likelihood estimate |
| --- | --- | --- |
| b_0_ | 2789.468 | 0.02040069 |
| b_1_ | 2840.114 | 0.33962388 |
| A | 2782.203 | 0.51442670 |
| B | 2801.693 | 0.37756892 |
| E | 3674.882 | 2.63292393 |

|  | Mean | 2.5% | 25% | 50% | 75% | 97.5% |
| --- | --- | --- | --- | --- | --- | --- |
| b_0_ | 0.020402 | 0.020353 | 0.020385 | 0.020402 | 0.020419 | 0.020455 |
| b_1_ | 0.339768 | 0.328518 | 0.335814 | 0.339738 | 0.343670 | 0.351078 |
| A | 0.514716 | 0.479802 | 0.502091 | 0.514325 | 0.526985 | 0.552013 |
| B | 0.378418 | 0.357158 | 0.371195 | 0.378343 | 0.385633 | 0.399907 |
| E | 2.644926 | 2.488146 | 2.590383 | 2.639954 | 2.697135 | 2.818411 |

### Model fit of preterm hospitalisation predictions to observed data


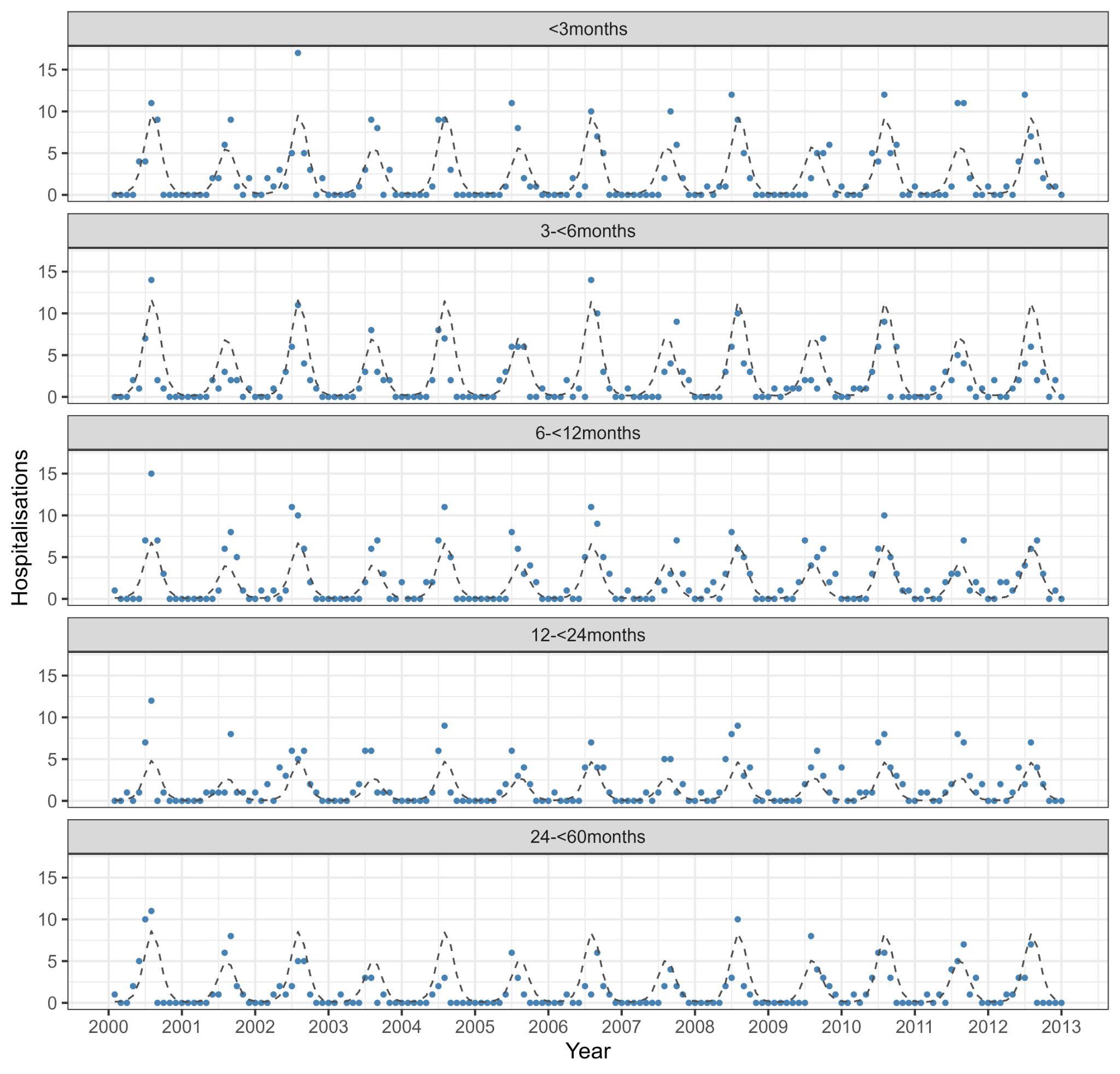
**Figure S4:** Comparison of the model estimated RSV hospitalisations for the preterm risk-group to the observed time series of the five age groups used to fit the model. The observed hospitalisations are shown with dots and the dashed line is the model output representing estimated hospitalisations.

## Age-to-risk

**Table S2:**

|  | Term | | Preterm | |
| --- | --- | --- | --- | --- |
| Age (months) | Risk (first exp) | Risk (sub exp) | Risk (first exp) | Risk (sub exp) |
| 0 | 0.5294 (100%) | 0.1059 | 1 (100%) | 0.2799 |
| 1 | 0.3677 (69.4%) | 0.0735 | 0.9720 (97.2%) | 0.1944 |
| 2 | 0.2568 (48.5%) | 0.0514 | 0.6788 (67.9%) | 0.1358 |
| 3 | 0.1807 (34.1%) | 0.0361 | 0.4778 (47.8%) | 0.0956 |
| 4 | 0.1286 (24.3%) | 0.0257 | 0.3400 (34.0%) | 0.0680 |
| 5 | 0.0929 (17.5%) | 0.0186 | 0.2456 (24.6%) | 0.0491 |
| 6 | 0.0684 (12.9%) | 0.0137 | 0.1808 (18.1%) | 0.0362 |
| 7 | 0.0516 (9.7%) | 0.0103 | 0.1364 (13.6%) | 0.0273 |
| 8 | 0.0401 (7.6%) | 0.0080 | 0.1060 (10.6%) | 0.0212 |
| 9 | 0.0322 (6.1%) | 0.0064 | 0.0851 (8.5%) | 0.0170 |
| 10 | 0.0268 (5.1%) | 0.0054 | 0.0708 (7.1%) | 0.0142 |
| 11 | 0.0231 (4.4%) | 0.0046 | 0.0610 (6.1%) | 0.0122 |
| 12 | 0.0205 (3.9%) | 0.0041 | 0.0543 (5.4%) | 0.0109 |

Note: Min risk for term is 0.015, min risk for preterm is 0.040

## Average age of hospitalisation


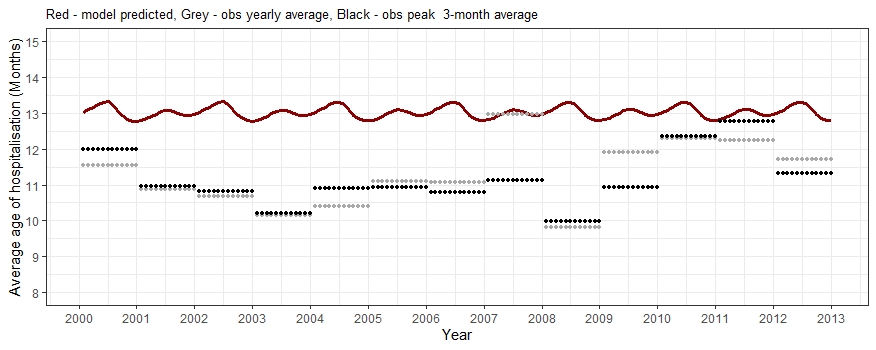


**Figure S5:** The red line shows the average age (in months) each month of model-predicted hospitalisations of children under 5 years of age. The grey dotted line shows the yearly average of observed hospitalisations of under 5-year-old children, and the black dotted line shows the average of observed hospitalisations of children under 5 during the months June - August for each year.

## Sensitivity testing

### C - minimum risk parameter

**Table S3:** The log likelihood values associated with different assumptions for C, the minimum average risk of hospitalisation for term infants, and the percentage change from the minimum log likelihood over the range of C values considered. The greyed row indicates the C value set in the model.

| **C** | **Log likelihood** | **% change from min** |
| --- | --- | --- |
| 0.005 | 12038.11 | 487.62 |
| 0.01 | 12694.18 | 519.65 |
| 0.015 | 12778.10 | 523.74 |
| 0.02 | 12602.05 | 515.15 |
| 0.025 | 12273.58 | 499.11 |
| 0.03 | 11843.53 | 478.12 |
| 0.035 | 11340.30 | 453.56 |
| 0.04 | 10781.50 | 426.28 |
| 0.045 | 10178.86 | 396.86 |
| 0.05 | 9540.63 | 365.71 |
| 0.055 | 8872.87 | 333.11 |
| 0.06 | 8180.12 | 299.30 |
| 0.065 | 7465.94 | 264.44 |
| 0.07 | 6733.15 | 228.67 |
| 0.075 | 5984.01 | 192.10 |
| 0.08 | 5220.38 | 154.82 |
| 0.085 | 4443.82 | 116.92 |
| 0.09 | 3655.63 | 78.44 |
| 0.095 | 2856.91 | 39.46 |
| 0.1 | 2048.62 | 0 |

### D - prior exposure scaling parameter

**Table S4:** The log likelihood values associated with different assumptions for D, the scaling of risk of hospitalisation for those who have experienced a prior RSV infection, and the percentage change from the minimum log likelihood over the range of C values considered. The greyed row indicates the D value set in the model.

| **D** | **Log likelihood** | **% change from min** |
| --- | --- | --- |
| 0 | 12834.55 | 3.01 |
| 0.05 | 12821.58 | 2.90 |
| 0.1 | 12807.82 | 2.79 |
| 0.15 | 12793.32 | 2.67 |
| 0.2 | 12778.10 | 2.55 |
| 0.25 | 12762.21 | 2.43 |
| 0.3 | 12745.68 | 2.29 |
| 0.35 | 12728.53 | 2.15 |
| 0.4 | 12710.79 | 2.01 |
| 0.45 | 12692.49 | 1.87 |
| 0.5 | 12673.66 | 1.71 |
| 0.55 | 12654.31 | 1.56 |
| 0.6 | 12634.46 | 1.40 |
| 0.65 | 12614.14 | 1.24 |
| 0.7 | 12593.37 | 1.07 |
| 0.75 | 12572.15 | 0.90 |
| 0.8 | 12550.52 | 0.73 |
| 0.85 | 12528.47 | 0.55 |
| 0.9 | 12506.04 | 0.37 |
| 0.95 | 12483.22 | 0.19 |
| 1 | 12460.04 | 0 |
